# HIV specific Th1 responses are altered in Ugandans with *HIV and Schistosoma mansoni* coinfection

**DOI:** 10.1186/s12865-023-00554-3

**Published:** 2023-08-29

**Authors:** Andrew Ekii Obuku, Jacqueline Kyosiimire Lugemwa, Andrew Abaasa, Moses Joloba, Song Ding, Justin Pollara, Guido Ferrari, Alexandre Harari, Giuseppe Pantaleo, Pontiano Kaleebu

**Affiliations:** 1 Medical Research Council/Uganda Virus Research Institute & London School of Hygiene and Tropical Medicine, Uganda Research Unit, Entebbe, Uganda; 2 School of Biomedical Sciences, Makerere University College of Health Sciences, Kampala, Uganda; 3 EuroVacc Foundation, Amsterdam, The Netherlands; 4 Department of Surgery, Duke University Medical Centre, Duke University, Durham, NC USA; 5 Department of Oncology, Lausanne University Teaching Hospital, Lausanne, Switzerland; 6 Division of Immunology and Allergy, Department of Medicine, Lausanne University Teaching Hospital, Lausanne, Switzerland; 7 Department of Clinical Research, London School of Hygiene & Tropical Medicine, London, UK

**Keywords:** HIV-1, Schistosomiasis, Immune responses, Down modulation

## Abstract

**Background:**

Fishing communities surrounding Lake Victoria in Uganda have HIV prevalence of 28% and incidence rates of 5 per 100 person years. More than 50% of the local fishermen are infected with *Schistosoma mansoni* (*S. mansoni*). We investigated the role of *S. mansoni* coinfection as a possible modifier of immune responses against HIV. Using polychromatic flow cytometry and Gran-ToxiLux assays, HIV specific responses, T cell phenotypes, antibody-dependent cell-mediated cytotoxic (ADCC) potency and titres were compared between participants with HIV-*S. mansoni* coinfection and participants with HIV infection alone.

**Results:**

*S. mansoni* coinfection was associated with a modified pattern of anti-HIV responses, including lower frequency of bifunctional (IFNγ + IL-2 − TNF-α+) CD4 T cells, higher overall CD4 T cell activation and lower HIV ADCC antibody titres, compared to participants with HIV alone.

**Conclusions:**

These results support the hypothesis that *S. mansoni* infection affects T cell and antibody responses to HIV in coinfected individuals.

**Supplementary Information:**

The online version contains supplementary material available at 10.1186/s12865-023-00554-3.

## Introduction

HIV infection has ravaged the population of sub Saharan Africa, creating demographic imbalance [1]. Sub Saharan Africa still has the highest proportion of HIV infected individuals. Indeed, two out of three new HIV infections in 2015 occurred in this sub region [2]. Although the HIV incidence in the sub region has stabilized, the prevalence is increasing as new infections occur daily and the provision of antiretroviral therapy is scaled up, improving survival [2].

However, the prevalence and incidence of HIV varies between populations. For example, HIV prevalence among sex workers is 12 times greater than the HIV prevalence among the general population [3]. Fishing communities in developing countries have HIV prevalence rates between 4 and 14 times higher than the national average [4]. Results from some fishing communities surrounding Lake Victoria in Uganda show a prevalence of 28% and incidence of 5% [5, 6].

Fishermen have enormous challenges of access to health services including provision of ART [7], yet ART has been shown to delay progression to AIDS. Indeed, fishermen are five times more likely to die of AIDS related illness than farmers in the Lake Victoria region [8].

However, few studies have investigated the reasons why the fishermen are more likely to die of AIDS related illness. In the Lambu fishing community of Lake Victoria, *S. mansoni* was the most common helminth infection identified. Although the number of deaths as a result of schistosomiasis alone is low, there is a great deal of unacknowledged morbidity [9]. Among the possible subtle effects of schistosomiasis and *S. mansoni* infection, an unresolved question is the extent to which schistosomiasis and *S. mansoni* infection influences the host response to other infections, such as HIV, and how this affects the rate of progression to AIDS.

Although few studies have directly investigated the association between *S. mansoni* infection and HIV progression to AIDS, multiple studies have shown that the plasma HIV RNA level is predictive of both HIV disease progression [10, 11] and risk of transmission of HIV to sexual partners [12] and that the treatment of *S. mansoni* infection with praziquantel (PZQ) reduced the rate of CD4 T cell decline in previously HIV and *S. mansoni* coinfected participants [13]. While treatment for schistosomiasis is clearly not a substitute for antiretroviral therapy (ART), it might slow HIV disease progression.

There are different categories of CD4 T cells including T helper (Th)1, Th2, Th17, Th22 and regulatory T cells (T reg). Th1 and Th2 are reciprocally inhibitory [14]. Since HIV is a predominantly Th1 phenotype inducing pathogen and *S. mansoni* is a predominantly Th2 inducing pathogen [14–16], we hypothesized that coinfection with *S. mansoni* infection impairs the potency of HIV specific responses and might enhance the rate of progression to AIDS.

## Results

### Bio-medical characteristics of study participants

Forty HIV + SM + and 37 HIV + SM − participants were selected. Sex distribution differed between the groups: 4/40 (10%) of HIV + SM + participants were females compared with 33/37 (89%) of HIV + SM − participants (Table 1). The mean age for both HIV + SM + and HIV + SM − participants was 30 years. The baseline mean plasma viral load among the HIV + SM + participants was higher than HIV + SM − participants {74,233 (range 0-327,900) copies per ml for HIV + SM +  and 57,662 (range 31–395,850) copies per ml for HIV + SM − participants p = 3.86 × 10^− 01^}; the baseline mean CD4 + T cell count among the HIV + SM + participants was slightly higher than HIV + SM − participants {667 cells (range 361-1,380)/µl of blood for HIV + SM + participants and 605 cells (range 354-1,227)/µl of blood for HIV + SM − participants p = 2.63 × 10^− 01^} (Table 1). Because cell numbers were limited in the samples available, the various assays of interest were conducted in subgroups of these participants, as shown in Table 1.

**Table 1:** Biomedical characteristics of participants whose PBMC were used in the immunology study

	HIV + SM+			HIV + SM−		
	Female	Male	Overall	Female	Male	Overall
No. of individuals (n)	4	36	40	33	4	37
Mean age in years (range)	30 (18–40)	30 (19–48)	30 (18–48)	29 (20–43)	38 (28–51)	30 (20–51)
Mean CD4 count per µl of blood (range)	924 (439-1,255)	638 (361-1,380)	667 (361-1,380)	627 (354-1,227)	419 (372–532)	605 (354-1,227)
Mean HIV RNA viral load copies per ml of plasma (range)	7,331 (21–28,084)	81,666 (0-327,900)	74,233 (0-327,900)	57,545 (31–395,850)	58,632 (6,873 − 133,862)	57,662 (31–395,850)

### Association of ***S. mansoni*** coinfection with T cell polyfunctionality in responders to HIV GAG

To investigate whether *S. mansoni* infection was associated with T cell polyfunctionality, we compared the proportion of responders to two HIV GAG PTE peptide pools between HIV + SM +  and HIV + SM − groups (response rates). Sixteen HIV + SM +  and 16 HIV + SM − participants were examined. The proportion of responders was high among, and not significantly different between, HIV + SM +  and HIV + SM − participants tested (Table S1).

**Fig. 1:**
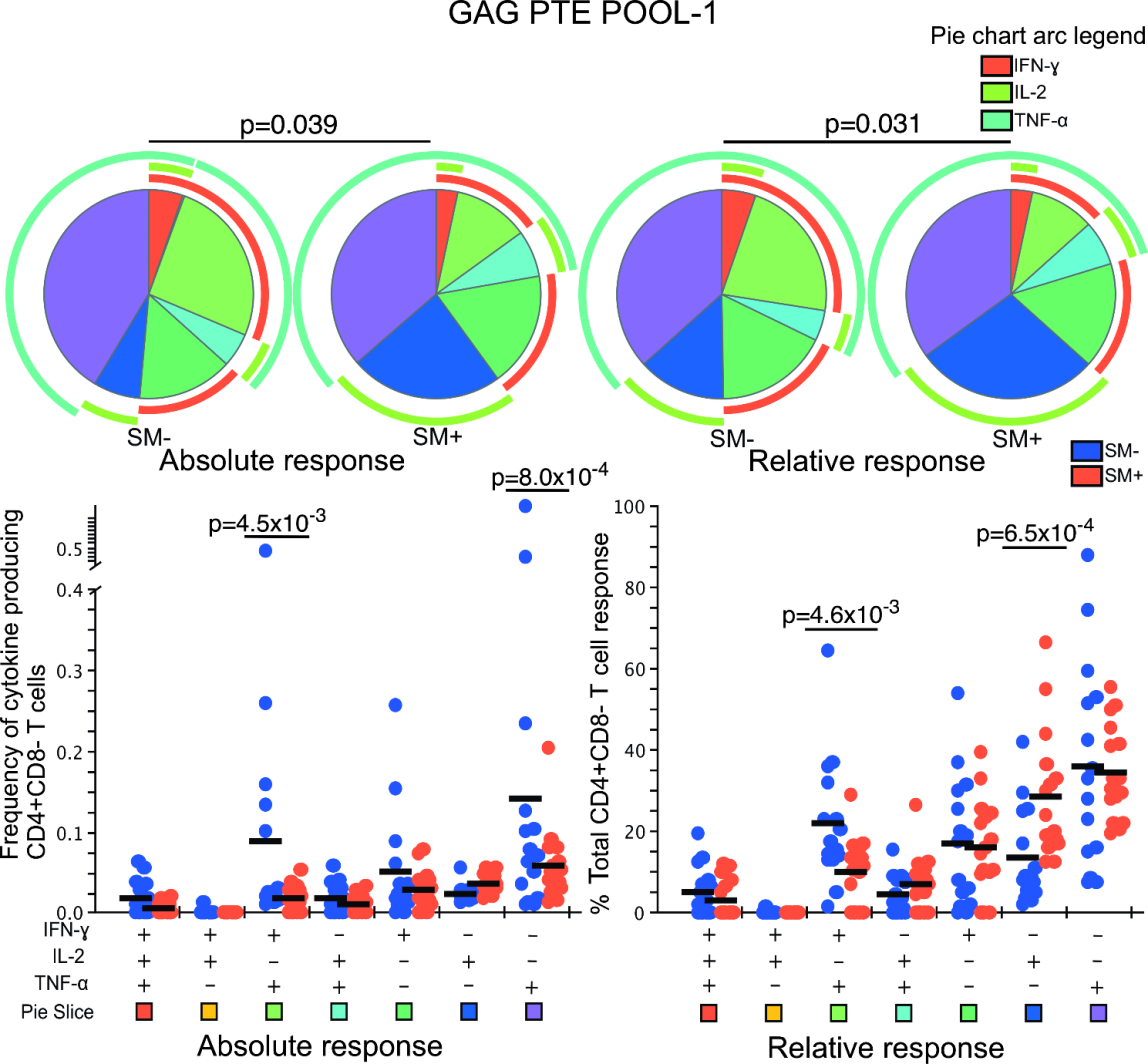
Polyfunctionality of CD4 T cell cytokine responses after GAG PTE POOL-1 stimulation. All possible combinations of positive responses are shown on the x-axis whereas frequencies of cytokine producing cells (absolute response) or percent of total CD4 T cell response (relative response) are shown on the y-axis of the scatter plots. The horizontal line shows the mean. Student’s t-test and Holm-Šídák correction for multiple comparisons test was used to compare the mean response for each CD4 T cell subset between HIV + SM+ (n = 16) and HIV + SM− (n = 15) responders. Only significant values are shown. To compare the pattern of representations of subsets of T cells, a partial permutation test was performed by Monte Carlo simulation with 10,000 iterations. A slice on a pie chart represents a Boolean gate defined subset of CD4 T cells secreting a single cytokine or a combination of cytokines. The size of a slice on the pie chart is the mean response from the scatter plot as a proportion of the overall response for each overlaid group. The colour of the squares at the bottom of the scatter plots matches that of the pie chart slices that correspond to each Boolean subset. The arcs around the pie charts indicate the production of individual cytokines over the Boolean subsets

**Fig. 2:**
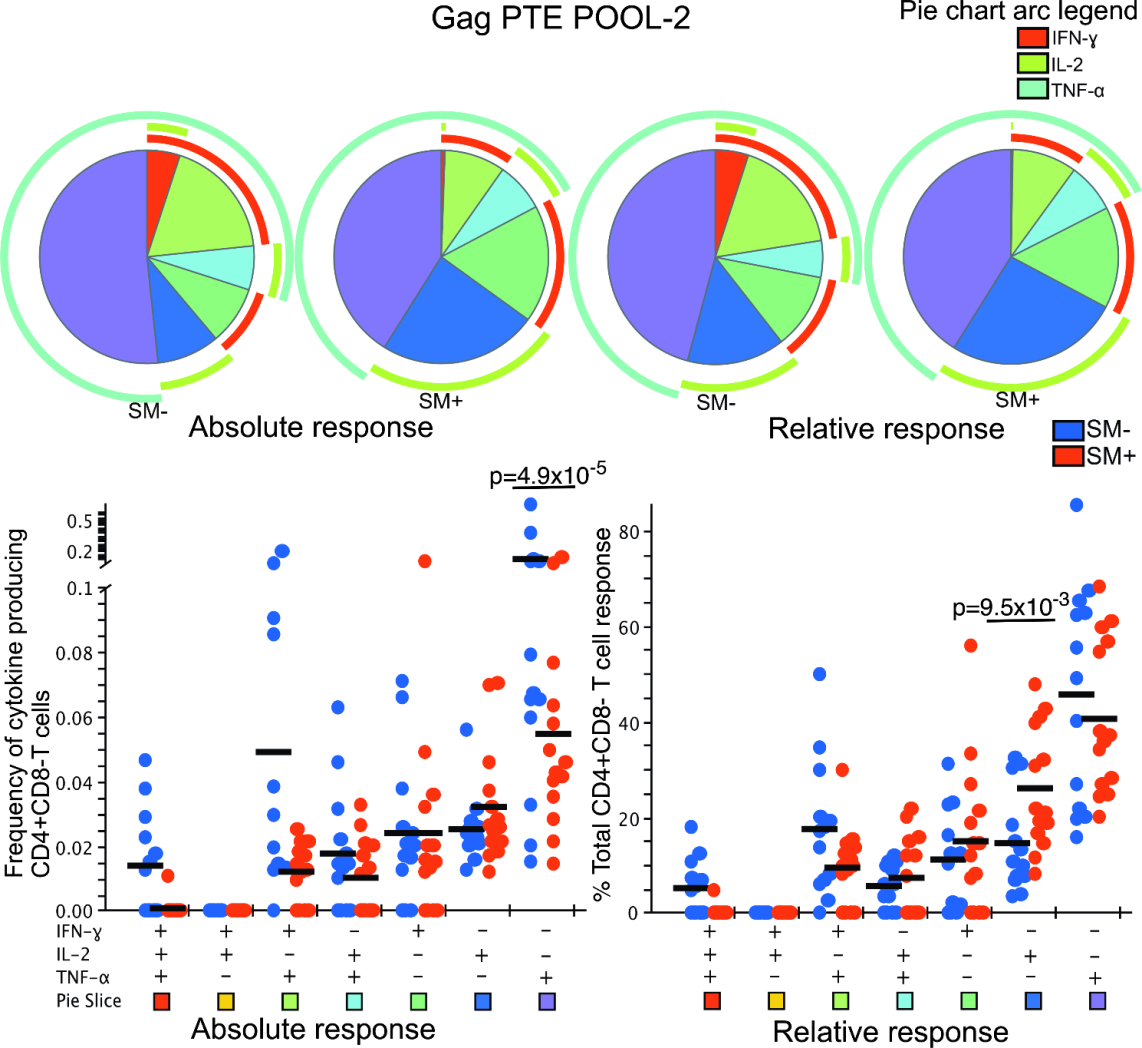
Polyfunctionality of CD4 T cell cytokine responses after GAG PTE POOL-2 stimulation. All possible combinations of positive responses are shown on the x-axis whereas frequencies of cytokine producing cells (absolute response) or percent of total CD4 T cell response (relative response) are shown on the y-axis of the scatter plots. The horizontal line shows the mean. Student’s t-test and Holm-Šídák correction for multiple comparisons test was used to compare the mean response for each CD4 T cell subset between HIV + SM+ (n = 13) and HIV + SM− (n = 14) responders. Only significant values are shown. To compare the pattern of representations of subsets of T cells, a partial permutation test was performed by Monte Carlo simulation with 10,000 iterations. A slice on a pie chart represents a Boolean gate defined subset of CD4 T cells secreting a single cytokine or a combination of cytokines. The size of a slice on the pie chart is the mean response from the scatter plot as a proportion of the overall response for each overlaid group. The colour of the squares at the bottom of the scatter plots matches that of the pie chart slices that correspond to each Boolean subset. The arcs around the pie charts indicate the production of individual cytokines over the Boolean subsets

We then examined the absolute and relative (normalised) frequencies of T cell functionality among the responders. In response to GAG PTE POOL-1 stimulation, the mean absolute frequency of IFN-γ + IL-2 − TNF-α+ {(0.09% (SM−) Vs 0.02% (SM+)} and IFN-γ − IL-2 − TNF-α+ {0.14% (SM−) Vs 0.06% (SM+)} CD4 T cells was significantly higher in the HIV + SM − compared to HIV + SM + responders (p = 4.5 × 10^− 3^ and p = 8.0 × 10^− 4^, respectively) (Fig. 1). When the normalised data were compared, the contribution of IFN-γ + IL-2 − TNF-α + subset to the overall response was higher among the HIV + SM − responders (22% (SM−) Vs 10% (SM+); p = 4.6 × 10^− 3^) (Fig. 1). However, among the HIV + SM + responders, the IFN-γ − IL-2 + TNF-α − subset contributed more to the overall response ((14% (SM−) Vs 28% (SM+) p = 6.5 × 10^− 4^)(Figs. 1 and 2 relative responses). Overall, the pattern of representation of CD4 T cell subsets in response to GAG PTE POOL-1 was significantly different between HIV + SM– and HIV + SM +  responders (p = 3.9 × 10^− 02^ absolute response and p = 3.1 × 10^− 02^ relative response) with monofunctional responses contributing more towards the overall response among the HIV + SM+ (Fig. 1 pie charts).

Similarly, a significant difference in the absolute response was observed in response to GAG PTE POOL-2 (Fig. 2). Specifically, the mean absolute frequency of IFN-γ − IL-2 − TNF-α+ {0.14% (SM−) Vs 0.06% (SM+)} CD4 T cells was higher in the HIV + SM − compared to HIV + SM + responders (p = 4.9 × 10^− 5^). In the relative comparison for GAG PTE POOL-2, the IFN-γ − IL-2 + TNF-α − subset contributed more to the overall response {(15% (SM−) Vs 26% (SM+) p = 9.5 × 10^− 3^} among the HIV + SM+ (Fig. 2).

Single cytokine producing CD4 T cells from HIV + SM + responders contributed over 75% of overall cytokine responses compared to approximately 50% from HIV + SM − responders. This pattern was similar for the relative responses. HIV specific CD4 dual and triple functional cells from HIV + SM + responders contributed to less than 12% of total response whereas those from HIV + SM − responders contributed almost 30% of total response (Figs. 1 and 2 pie charts).

In response to GAG PTE POOL-1 stimulation, there was no CD8 T cell subset that was significantly different between HIV + SM + and HIV + SM − participants (S6 Fig).

**Fig. 3:**
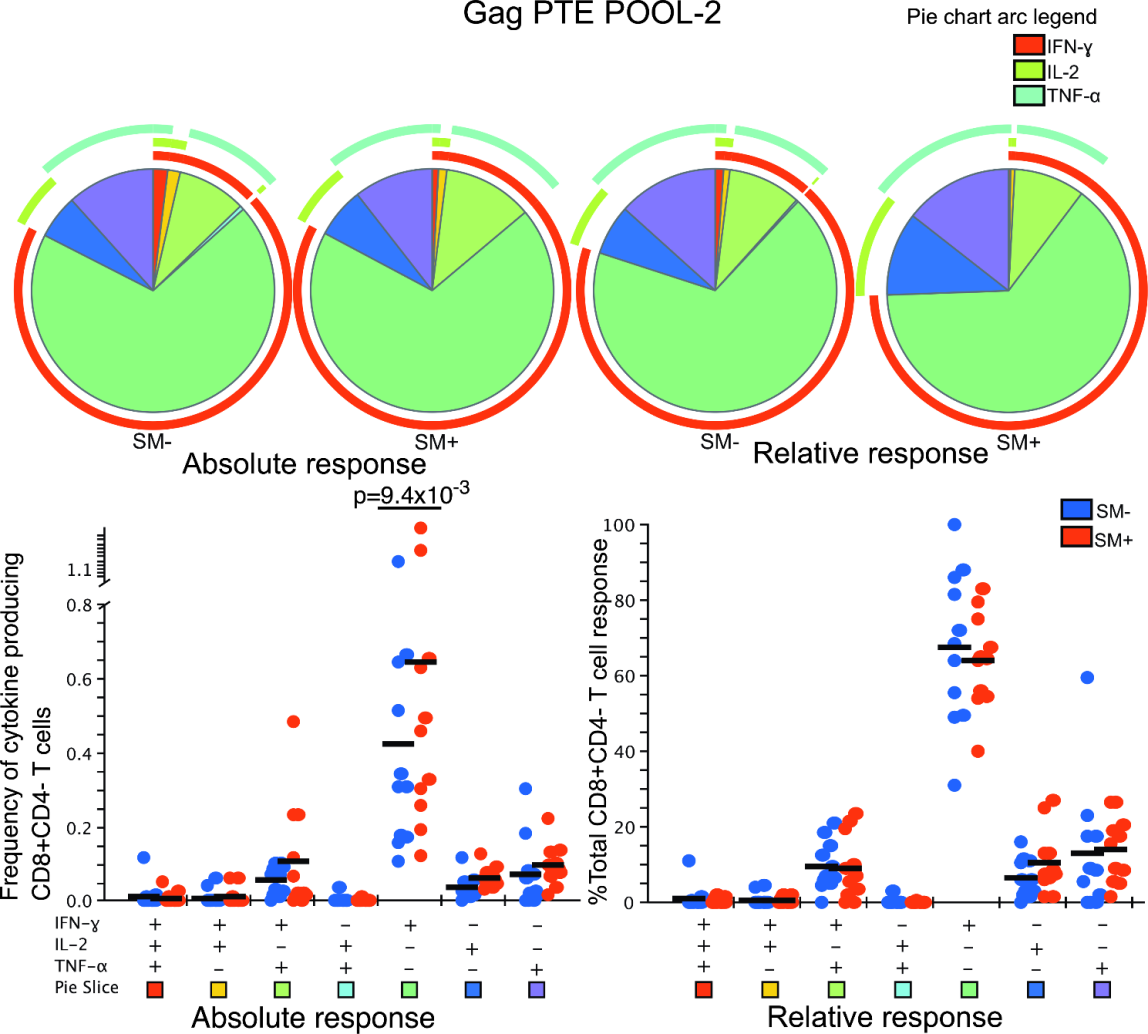
Polyfunctionality of CD8 T cell cytokine responses after GAG PTE POOL-2 stimulation. All possible combinations of positive responses are shown on the x-axis whereas frequencies of cytokine producing cells (absolute response) or percent of total CD4 T cell response (relative response) are shown on the y-axis of the scatter plots. The horizontal line shows the mean. Student’s t-test and Holm-Šídák correction for multiple comparisons test for was used to compare the mean response for each CD4 T cell subset between HIV + SM+ (n = 11) and HIV + SM− (n = 11) responders. Only significant values are shown. To compare the pattern of representations of subsets of T cells, a partial permutation test was performed by Monte Carlo simulation with 10,000 iterations. A slice on a pie chart represents a Boolean gate defined subset of cells secreting a single cytokine or a combination of cytokines. The size of a slice on the pie chart is the mean response from the scatter plot as a proportion of the overall response for each overlaid group. The colour of the squares at the bottom of the scatter plots matches that of the pie chart slices that correspond to each Boolean subset. The arcs around the pie charts indicate the production of individual cytokines over the Boolean subsets

The mean absolute frequency of IFN-γ + IL-2 − TNF-α − CD8 T cells in response to GAG PTE POOL-2 stimulation was significantly higher among HIV + SM + compared to HIV + SM − responders {mean = 0.42% (SM−) Vs 0.64% (SM+)} p = 9.4 × 10^− 3^ (Fig. 3). However, the statistical significance was not retained when normalized data were compared. The frequency of responding CD8 T cells was higher than the frequency of responding CD4 T cells.

### Association of ***S. mansoni*** coinfection with MFI of HIV specific CD4 and CD8 T cells in HIV + responders.

We then investigated whether *S. mansoni* infection was associated with the intensity of IL-2, TNF-α or IFN-γ produced by HIV specific CD4 and CD8 T cells on a per cell basis. The CD4 IFN-γ and TNF-α MFI in response to GAG PTE POOL-1 and GAG PTE POOL-2 stimulations were significantly lower among the HIV + SM + responders compared to HIV + SM − responders (Table 2). However, the CD8 T cells IFN-γ, IL-2 or TNF-α MFI in response to GAG PTE POOL-1 and GAG PTE POOL-2 stimulations were not significantly different between HIV + SM + and HIV + SM − responders (S2 Table).

**Table 2:** CD4 Mean median fluorescence intensity between HIV + SM + and HIV + SM−, among responders

Cytokine	Mean MFI SM− (n)	Mean MFI SM+ (n)	p-value*	Stimulant
IFN-γ	1530 (15)	925 (16)	1.5 × 10^− 02^	GAG PTE POOL-1
IL-2	452 (15)	339 (16)	1.3 × 10^− 01^	GAG PTE POOL-1
TNF-α	2282 (15)	1448 (16)	8.0 × 10^− 06^	GAG PTE POOL-1
IFN-γ	1565 (13)	843 (14)	7.1 × 10^− 03^	GAG PTE POOL-2
IL-2	399 (13)	276 (14)	1.2 × 10^− 01^	GAG PTE POOL-2
TNF-α	2137 (13)	1302 (14)	2.7 × 10^− 05^	GAG PTE POOL-2

* Student t test was used in the comparison between HIV + SM + and HIV + SM − responders

### Association of ***S. mansoni*** coinfection with Th profiles of CD4 T cells in HIV + participants

Having observed lower frequencies of bifunctional (IFN-γ + IL-2 − TNF-α+) CD4 T cells in the experiments above, we hypothesized that the “missing” HIV specific Th1 cells might have developed another Th phenotype characterized by production of different cytokines. To test this hypothesis we used the Th1/Th2/Th9/Th10/Th17 phenotype panel to stain CD4 T cells stimulated with GAG PTE POOL-1, -2 or p24 or SEB.

In response to GAG PTE POOL-1 (HIV + SM + n = 9; HIV + SM − n = 5) and SEB (HIV + SM + n = 14; HIV + SM − n = 10) stimulations, the HIV + SM − participants had higher mean ratios of frequency of IL-10 secreting CD4 T cells or frequency of Th2 (IL-4/5/13 secreting CD4 T cells) or frequency of Th9 (IL-9 secreting CD4 T cells) or frequency of Th17 (IL-17 A secreting CD4 T cells) to Th1 (IFN-γ or TNF-α secreting cells) compared to HIV + SM + except for the Th2 to Th1 ratio in response to GAG PTE POOL-1 stimulation. However, in response to p24 (HIV + SM + n = 5; HIV + SM − n = 4) and GAG PTE POOL-2 (HIV + SM + n = 9; HIV + SM − n = 5) stimulations, the mean ratios of Th2, Th9, IL-10 producing CD4 T cells and Th17 to Th1 were higher in the HIV + SM + compared to HIV + SM − participants. All these differences did not approach statistical significance (Fig S7).

In summary, there was no evidence to support the hypothesis that significant skewing of HIV specific Th1 to HIV specific Th2, Th9, Th10 or Th17 occurred in HIV + SM + participants.

### Association of ***S. mansoni*** coinfection with CD4 T cell activation in HIV + SM + participants

**Fig. 4:**
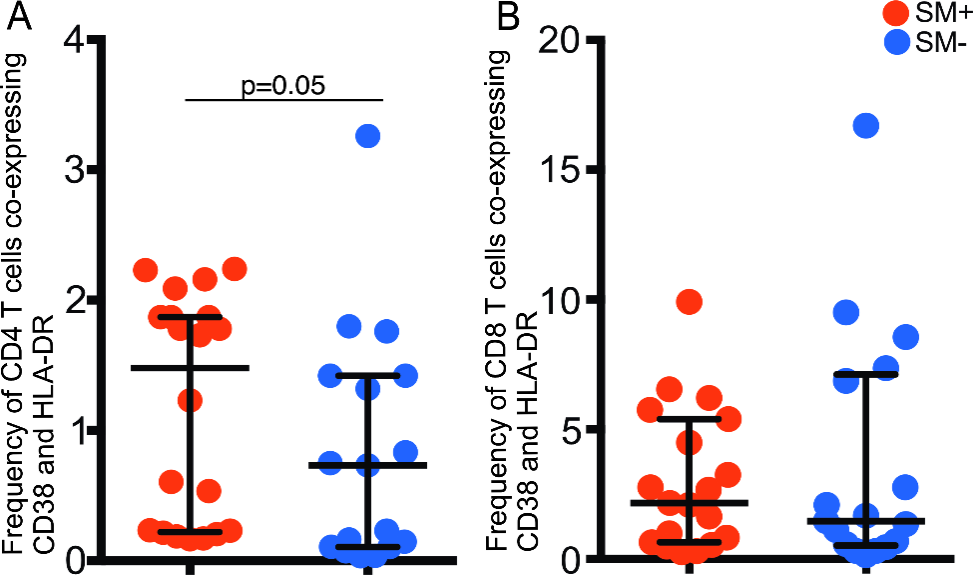
Association of T cell activation and *S. mansoni* infection in HIV + SM+. Frequency of (A) CD4 or (B) CD8 T cells coexpressing HLA-DR and CD38 in HIV + SM+ (red dots, n = 19) and HIV + SM− (blue dots, n = 17). The Mann Whitney test was used to compare the mean ranks of activated CD4 and CD8 T cells in HIV + SM + and HIV + SM−. The horizontal line shows the median and the vertical line shows the interquartile range. Only significant p values are shown

Since T cell activation (defined as co-expression of HLA-DR and CD38) occurs both in HIV and *S. mansoni* infections, we hypothesized that T cell activation might be enhanced in coinfections. Samples from 19 HIV + SM + and 17 HIV + SM − participants were examined. Indeed, the HIV + SM + participants had higher frequencies of activated CD4 T cells compared to HIV + SM − participants (median = 1.48% (SM+) Vs 0.73% (SM−) (p = 5.0 × 10^− 02^) while there was no significant association for CD8 T cells (median = 2.16% (SM+) Vs (median = 1.46% (SM−) (p = 8.5 × 10^− 02^) (Fig. 4).

However, the frequency of CD4 or CD8 T cells expressing CD38, CD160, CD150, CD244, CD279 or HLA-DR was not significantly different between HIV + SM + and HIV + SM − participants (Table S3).

### Association of ***S. mansoni*** coinfection with CD8 T cell cytotoxic potential in HIV + SM + responders

Owing to the importance of cytotoxicity in the control of HIV infection, we investigated whether *S. mansoni* infection was associated with differences in the cytotoxic potential of HIV specific CD8 T cells.

**Fig. 5:**
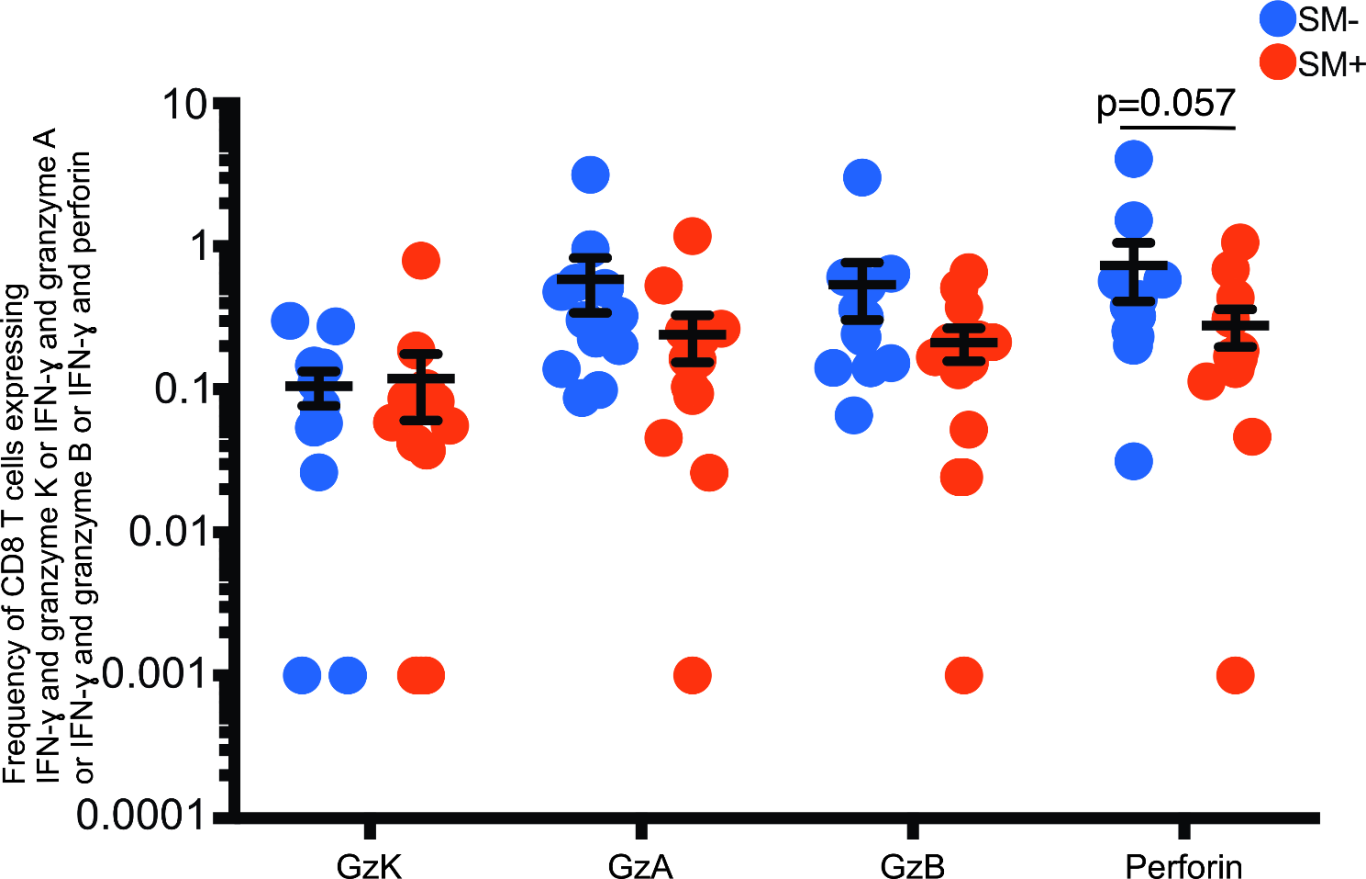
Association of frequency of CD8 T cells expressing cytolytic granules and *S. mansoni* infection among HIV + SM+ coinfected individuals. Comparison of the frequency of Granzymes K, A, B & perforin expressing CD8 T cells between HIV + SM + and HIV + SM − in response to separate GAG PTE POOL-1 and 2 stimulations. GAG PTE POOL-1 and GAG PTE POOL-2 responses were added together in the analysis. The horizontal line shows the mean and the vertical line shows the 95% confidence interval. Student’s t test and the Holm-Šídák correction for multiple comparisons were used to compare the mean frequencies of CD8 T cells with cytotoxic potential between HIV + SM + and HIV + SM−. Twelve HIV + SM + and 12 HIV + SM − participants were stimulated with GAG PTE POOL-1 and GAG PTE POOL-2.

The frequency of GAG PTE POOL-1 and − 2 specific CD8 T cells co-expressing IFN-γ and perforin (mean = 0.74% (SM−) Vs 0.28% (SM+) was somewhat lower in the HIV + SM + compared to HIV + SM − responders (p = 5.7 × 10^− 02^) (Fig. 5). In addition, a trend towards lower frequencies of GAG PTE POOL-1 and − 2 specific CD8 T cells expressing granzymes A, B and K was observed among the HIV + SM + participants compared to the HIV + SM − participants (Fig. 5).

### Association of ***S. mansoni*** coinfection with ADCC potency and titre in HIV + participants

**Fig. 6:**
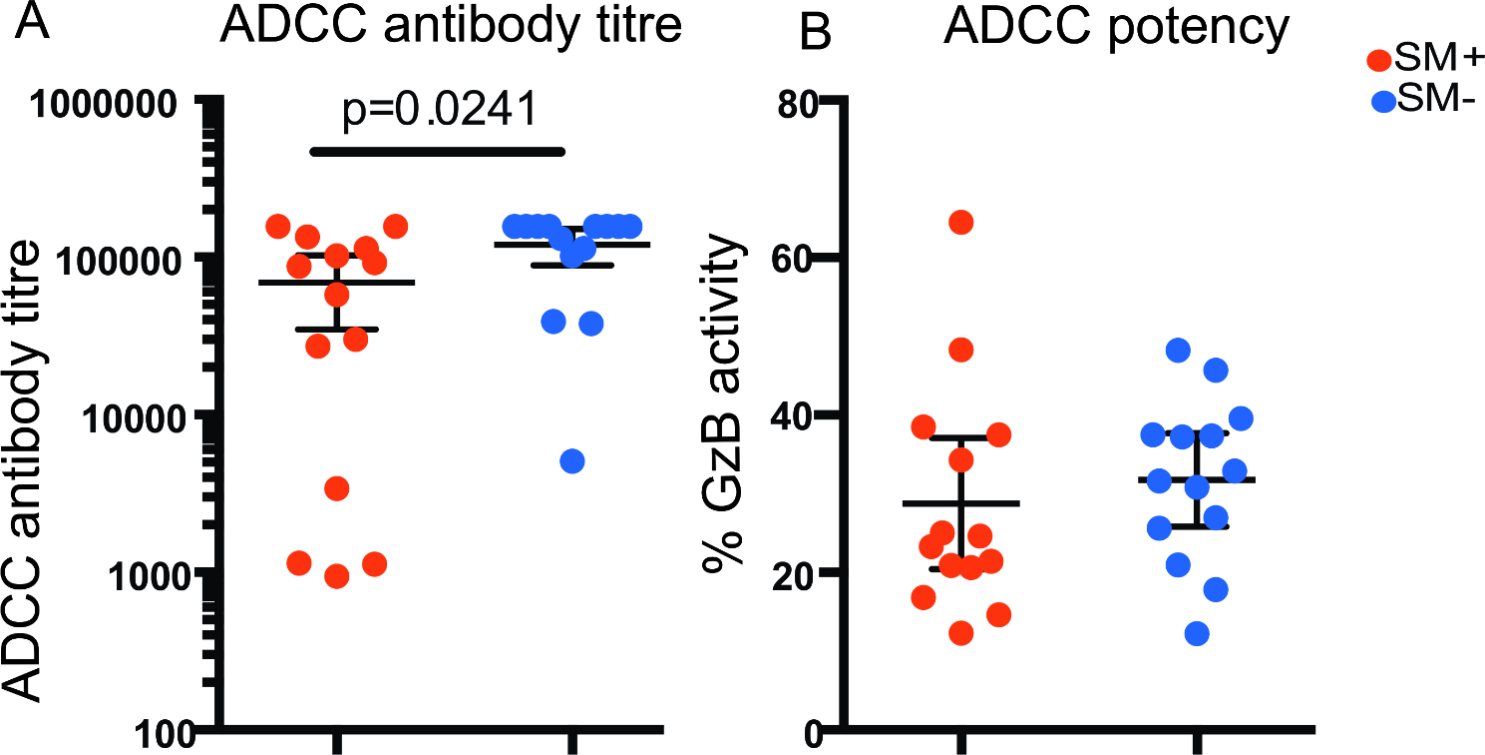
* S. mansoni* infection is associated with lower ADCC antibody titre among HIV + SM + participants. (A) ADCC antibody titre and (B) proportion of Granzyme B activity in target cells (%GzB) between HIV + SM+ (red dots n = 14) and HIV + SM− (blue dots n = 14). Student’s t test was used in the comparison. Only significant p values are shown. The middle horizontal line on the data points shows the mean and the two lines flanking the mean shows the 95% confidence interval

Since Th2 phenotype has been associated with help for antibody responses, we investigated the association of *S. mansoni* infection with HIV specific ADCC potency and titres using the Gran-ToxiLux assay. The A1953 specific ADCC antibody titre was significantly higher in the HIV + SM − compared to the HIV + SM + participants (p = 2.4 × 10^− 02^) (Fig. 6A). However, the potency of A1953 ADCC antibodies was not significantly different between HIV + SM + and HIV + SM− (Fig. 6B).

## Materials and methods

### Study groups

HIV positive individuals were recruited to a “Schistosomiasis Intervention Study” (SIS) in the Lambu, Kassa, Mitondo, Kisuku, Kabasese, Kachanga, Bukakata, Bukibonga and Kaziru villages on the shores of Lake Victoria. Samples were selected for this immunological substudy from participants with and without *S. mansoni* co-infection. The HIV + only infected participants were the reference group. Participants were included if they were adults (aged 18 years or above), HIV infected, not on ART, with CD4 T cell counts above 350 cells per microliter of blood. Participants were classified as *S. mansoni* positive or negative based on 3 consecutive Kato Katz (KK) determinations of stool egg burden. Participants were excluded from this work if found to have any other intestinal helminth or parasitic infection on KK examination. HIV infection was determined by rapid diagnostic tests and followed by plasma viral load measurement. The duration of HIV and *S. mansoni* infections and their temporal relationship was not determined. Based on guidelines at the time of the study, participants were referred for HIV care and treated if CD4 counts fell below 350 cells per microliter of blood.

About 90 ml of blood was drawn at enrolment from each participant. Peripheral blood mononuclear cells (PBMC) were separated using ficoll histopaque 1077 (Sigma, Darmstadt, Germany) and cryopreserved in liquid nitrogen. Participants were treated with PZQ either twice within a week after enrolment (intensive treatment) or received a single standard treatment 12 weeks after enrolment.

### Flow cytometry staining assay

The cryo-preserved PBMC (1-2 × 10^6^) were thawed and rested for 6 hours and stimulated overnight in 1 ml of complete medium (RPMI (Sigma), 10% fetal bovine serum (FBS; PAA, Austria), 100µg/ml penicillin, 100 units/ml streptomycin (Lonza, Switzerland) in the presence of Golgiplug (1µl/ml, BD), (and Golgistop (1µl/ml, BD) for the cytotoxic potential panel) and anti-CD28 (0.5 µg/ml, BD) and 1 µg/ml of potential T cell epitopes (PTE) peptide pools (GAG PTE POOL-1 and GAG PTE POOL-2) [17] or 5µg/ml of p24 protein (protein science corporation). Staphylococcus enterotoxin B (SEB; Sigma) stimulation (200ng/ml) was used as a positive control. Medium alone was used as a negative control. At the end of the stimulation period, cells were stained for 20’ at 4 °C using Aqua LIVE/DEAD cell stain (Invitrogen), fixed and permeabilised (Cytofix/Cytoperm, BD) at room temperature for 20 min and then stained at room temperature for 20 min with anti human CD3 APC-H7, CD4 PE-CF594, CD8 Pacific Blue, TNF-α PE-Cy7, IL-2 PE, IFN-γ APC (from BD, Biosciences) and Perforin FITC (from GenProbe) (Th1 phenotype panel) or anti human CD3 APC-H7, CD4 PE-CF594, IFN-γ Alexa 700, TNF-α PE-Cy7, IL-4 PE (from BD, Biosciences), CD45RA BV711, IL-2 PerCPCy5.5, IL-10 BV421, IL-17 A FITC, IL-5 PE, IL-13 PE, IL-9 Alexa Fluor 649 (from Biolegend) and CD8 eFluor 625NC (from eBiosciences) (Th1/Th2/Th9/Th10/Th17 phenotype panel) or anti human CD3 ECD (from Beckman Coulter), CD8 APC-H7, IFN-γ APC, Granzyme B Alexa Fluor 700 (from BD, Biosciences), Perforin PE, Granzyme A (from Biolegend), CD4 eFluor 650NC (from Bender MedSystems) and Granzyme K FITC (from Santa Cruz) (Cytotoxicity potential panel). The stained cells were washed twice with buffer containing 98% phosphate buffered saline and 2% FBS (P2). Cellfix (BD, San Diego, CA, USA) was added and 0.6-1 × 10^6^ lymphocyte-gated events were immediately acquired on an LSRΙΙ SORP analyser (BD, San Diego, CA, USA).

For staining with the positive/negative regulatory panel (surface staining), rested PBMC were stained for 20 min at 4 °C using Aqua LIVE/DEAD cell stain (Invitrogen). The PBMC were then stained at room temperature for a further 20 min with anti human CD3 APC-H7, CD4 FITC, CD279 PECy7, CD38 PE-CF594 (from BD, Biosciences), CD8 Pacific Blue, CD150 PE, CD160 Alexa Fluor 647, CD244 PE-Cy5.5 (from Biolegend) and HLA-DR Alexa Fluor 700 (from eBiosciences). After incubation, the stained cells were washed twice with P2. Cellfix (BD, San Diego, CA, USA) was added and 0.6-1 × 10^6^ lymphocyte-gated events were immediately acquired on an LSRΙΙ SORP (BD, San Diego, CA, USA) after completion of staining.

For staining with the T reg panel, rested PBMC were stained for 20 min at 4 °C using Aqua LIVE/DEAD cell stain (Invitrogen). The PBMC were then stained at room temperature for a further 20 min with anti human CD3 APC-H7, CD4 Pacific Blue, CD25 PE-Cy7 (from BD, Biosciences), CD8 PerCPCy5.5, CD127 BV711, GARP APC, CD39 FITC (from Biolegend) and CD45R0 (from Beckman Coulter). FoXP3 fixation and permeabilisation buffer was added and the cells were incubated for 15 min. After two washes, normal rat serum (from eBiosciences) was added and the cells were incubated for a further 15 min. FoXP3 antibody (from eBiosciences) was then added and the cells were incubated for 15 min after which they were washed. Cellfix (BD, San Diego, CA, USA) was added and 0.6-1 × 10^6^ lymphocyte-gated events were immediately acquired on the analyser after completion of staining.

Cytometer setup and tracking beads (BD, San Diego, CA, USA) were used in the calibration of the analyser daily and voltages determined by FACS DIVA version 6.0 during the running of the cytometer setup and tracking beads were used to run the participant samples.

Flowjo version 9.8.4 (Treestar, Ashland, OR, USA) was used in the analysis. To exclude doublets, data on forward scatter area and forward scatter height were used. Aqua positive cells were also excluded. S1-S5 Figs shows the gating strategy for the five flow cytometry panels.

### ADCC gran-toxilux assay

The ADCC Gran-ToxiLuX assay kit was purchased from OncoImmunin Inc. (California, USA). The assay procedure was done according to earlier publication (Pollara et al. 2011). Briefly, to generate the target cells, the CEM-NKRCCR5 cell line obtained from the NIH AIDS Research and reagent programme was infected with the A1953 variant of HIV-1 IIIB virus (kindly provided by Dr. Hoxie, University of Pennsylvania).

The effector cells were PBMC obtained from an HIV seronegative donor using leukapheresis, processed using ficoll-histopaque 1077 and cryopreserved within 6 hours of collection. The cells were later thawed and rested overnight at 2 × 10^6^cells/ml in R10 at 37 °C in 5% CO2 incubator. The cells were counted and viability determined to plate 10,000 viable target cells per condition. A minimum of 80% viability threshold was used.

The target cells were labeled with fluorescent target cell marker (TFL4) and viability marker (NFL1) for 15 min in a 37 °C water bath. After washing using 10ml of R10, the cells were counted using a Guava PCA and adjusted to have an effector to viable target cell ratio of 30:1. The effector and labelled target cells were then poured into a trough and mixed by pipetting. Seventy-five microliters of Granzyme B substrate was then added into each well of a 96 well V bottom plate and 50 µl of target/effector cell suspension was also added to each well and mixed. After 5 min incubation at room temperature, 25 µl of the diluted plasma or IgG preparations were added to the Granzyme B substrate and target/effector cell suspension in the well and incubated at room temperature for 15 min. The plates were incubated for one hour in a 5% CO2 incubator at 37 °C after centrifugation at 300 g for 1 min. The cells were washed twice with wash buffer and resuspended in 225 µl of wash buffer and acquired directly from the assay plate with the LSRΙΙ high throughput sampler following the manufacturer’s instructions.

The maximum %Granzyme B (%GzB) activity at any given dilution is referred to as potency and the ADCC mediating antibody titre in the plasma of the donor is obtained by interpolating the dilution at which the curve intersects the positive cut-off value after excluding the prozone area. The positive cut off value for the A1953 infected targets was set so that the fraction of HIV uninfected seronegative samples yielded less than 12% GzB activity.

### Binding antibody ELISA

Flat bottom 96-well 4HX Immulon plates (Thermo Scientific, NY, USA) were coated with 50 µl per well of 0.2 µg/ml of gp41 MN (Cat. No. 12,027), 0.2 µg/ml gp140 UG21 clade D (Cat. No. 12,065), 0.2 µg/ml gp140 UG37 clade A (Cat. No. 12,063) or 0.2 µg/ml gp140 SF162 (Cat. No. 12,026) clade B antigens in 0.1 M sodium bicarbonate coating buffer. Each antigen was coated on a separate plate. The antigens were obtained from the NIH AIDS Research and Reagent Program. The coated plates were incubated overnight at 4^o^C. The plates were washed four times with PBST (PBS with 0.03% Tween 20) and blocked with 150 µl per well of 1% marvel (skimmed milk powder in PBS with 0.03% Tween 20) for two hours at room temperature.

The blocking buffer was removed and 50 µl per well of diluted plasma samples (1/200) were added. The plasma samples were diluted with 0.1% dried skimmed milk (Marvel). The samples were incubated at room temperature for two hours covered with damp paper towel and polythene bag.

The plates were washed 6 times with wash buffer (PBS with 0.03% Tween 20) and 50 µl per well of biotinylated mouse anti-human IgG1 (BD Cat. No.555,869) or IgG3 (Merck Millipore Cat. No. 411,483) monoclonal antibody was added at a final concentration of 0.5 µg/ml. The plates were incubated at room temperature for one hour covered with damp paper towel and polythene bag.

The plates were washed 6 times with wash buffer (PBS with 0.03% Tween 20) and 50 µl of poly-HRP-streptavidin conjugate (Sanquin, The Netherlands) was added at 1/4000 dilution and incubated for one hour at room temperature.

One hundred microliters of o-phenylenediamine dihydrochloride substrate was added to each well and the reaction was stopped after 15 min with 25 µl of 2 M sulphuric acid. The plate wells’ absorbencies were read at 490 and 630 nm.

To obtain the antibody concentrations, the absorbency for each well measured at 490 nm were subtracted from the absorbency measured at 630 nm for the same well. The difference in absorbencies of the standards was plotted against the concentration to draw standard curves. Antibody concentrations were determined using standard curves and limits of quantitation calculated according to a 5-parameter logistic model. The myeloma proteins that served as standards were purchased from sigma Cat. No. I 5154 and I 5654.

### Data analysis

For the participants whose PBMC were stained with Th1 phenotype, three different parameters were compared:

1. Response rates: the number of responders was compared to the number of non-responders using Fishers exact test. A responder was defined as any participant with either IFN-γ or IL-2 or TNF-α response (% of cells) above the 95th percentile of unstimulated response after background (unstimulated control) subtraction.
2. Polyfunctionality: Boolean gated single cytokine responses were compared between HIV + SM + and HIV + SM − responders using Student’s t test.
3. Median Fluorescence Intensity (MFI): the MFI for each cytokine was compared between HIV + SM + and HIV + SM − responders using Student’s t test.

To analyse whether the skewing of Th1 to another Th phenotype occurred among the HIV + SM + participants, the ratio of frequency of IL-10 secreting CD4 T cells or frequency of Th2 (IL-4/5/13 secreting CD4 T cells) or frequency of Th9 (IL-9 secreting CD4 T cells) or frequency of Th17 (IL-17 A secreting CD4 T cells) to Th1 (IFN-γ or TNF-α secreting cells) was compared between HIV + SM + and HIV + SM − participants. To avoid divisions by 0, a fixed offset of 0.001 was added to all values before computing the ratios. Student’s t test and Holm-Šidák multiple test correction with an α of 0.05 (GraphPad Prism v6.h) was used for statistical comparisons.

To investigate the association of *S. mansoni* infection with the cytotoxic potential of HIV specific CD8 T cells, the frequency of GAG specific CD8 T cells (defined as producing IFN-γ in response to GAG stimulation) coexpressing Granzyme K, Granzyme A, Granzyme B or perforin was computed. Then, the frequency of CD8 T cells coexpressing IFN-γ and Granzyme K, IFN-γ and Granzyme A, IFN-γ and Granzyme B or IFN-γ and perforin from unstimulated cells was deducted from corresponding GAG PTE POOL-1 and GAG PTE POOL-2 stimulated cells to compute the net response. After this, the responses for each marker of cytotoxic potential in response to GAG PTE POOL-1 were added to those of GAG PTE POOL-2 to estimate the total response. The data from responders to both GAG PTE POOL-1 and GAG PTE POOL-2 were then transformed to log10 for normalization and normality was tested using Shapiro-Wilk normality test. Student’s t test was used to compare the means between HIV + SM + and HIV + SM − participants.

To investigate the association of *S. mansoni* infection with T cell activation, the frequency of T cells coexpressing HLA-DR and CD38 was also compared between HIV + SM + and HIV + SM − participants using Student’s t test. Holm-Šidák multiple test correction with an α of 0.05 was used to correct for multiple comparisons.

## Discussion

We investigated immunological differences between HIV-positive adults with and without *S. mansoni* coinfection in whom both infections were presumed to be of long standing. Coinfection was associated with a lower frequency of dual-function HIV specific CD4 T cells and lower CD4 IFN-γ and TNF-α production in responding cells. We did not find evidence of skewing from HIV specific Th2, Th9, Th17 cells, or of increased IL-10 producing HIV-specific CD4 T cells. HIV and *S. mansoni* coinfection was associated with higher overall levels of activated CD4 T cells. HIV and *S. mansoni* coinfection was also associated with a higher frequency of HIV-specific IFN-γ positive CD8 T cells, but these cells showed a lower frequency of perforin production. As well, *S. mansoni* coinfection was associated with lower A1953 specific ADCC titres.

Our cross-sectional study design makes it difficult to determine causation. Plasma HIV load was higher among HIV + SM + participants (significantly so in subsets studied for some assays) and, while this might be the result of *S. mansoni* coinfection [13], it may also be a chance finding and the higher viral load may have contributed to some of the effects observed. Sample numbers were small for some of the comparisons made, limiting study power. It was not possible to estimate the duration of HIV or of *S. mansoni* infection in this study, or to determine whether *S. mansoni* infection preceded HIV infection.

### HIV specific CD4 T cell responses

Our CD4 T cell response results suggest a more complex picture than the simplistic Th1 Vs Th2 or Th1 Vs T reg model of mutual exclusion, which has been widely proposed [14–16]. In this study, HIV + SM + responders displayed a lower frequency of IFN-γ + IL-2 − TNF-α + CD4 T cells, lower IFN-γ and TNF-α production amongst responders, and a higher proportion of single cytokine producing CD4 T cells within the overall response among the HIV + SM+. This is of concern because the frequency of triple and dual (IFN-γ/IL-2/TNF-α) producing CD4 T cells has been associated with better HIV control [18]. For example, CD4 and CD8 T cells which produce IFN-γ alone have limited capacity to develop into memory T cells compared with IL-2 or IL-2 + IFN-γ + producing cells [19–21]. Similarly, measles specific polyfunctional T cells have been shown to facilitate measles RNA clearance [22]. In our study, among the HIV + SM + participants who were analysed by Th1 panel, those with lower IFN-γ + IL-2 − TNF-α + CD4 T cells, had a significantly higher plasma viral load (p = 8.0 × 10^− 03^): whether this was the result, or the cause, of the different cell profiles remains to be determined.

Besides a reduction in frequency of TNF-α producing cells, the reduction in intensity of TNF-α production per cell (represented by reduced MFI) among the HIV + SM + responders may have functional consequences.

Despite the relatively higher contribution of the IFN-γ − IL-2 + TNF-α − subset to the overall CD4 T cell response among the HIV + SM + responders, there was no concordant increase in the frequency of T reg (S8 Fig): this might have been expected because IL-2 functions to maintain homeostasis and competitive fitness of Tregs [23]. Since we measured activated T reg, and not HIV specific T reg, it is possible that this bulk measurement of activated T reg could mask a selective increase of HIV specific T reg.

The findings of this study contrast with our earlier work on recently HIV infected individuals, among whom *S. mansoni* infection undoubtedly preceded HIV infection [24]. In the earlier study we found enhanced production of TNF-α in response to innate stimuli, and enhanced frequencies of HIV-specific IFN-γ + IL-2 − TNF-α + CD4 T cells (as well as IFN-γ + IL-2 − TNF-α- CD8 T cells) among *S. mansoni* co-infected individuals. One possible explanation of this difference is that the IFN-γ + IL-2-TNF-α + CD4 T cell subset might convert to monofunctional IFN-γ + and TNF-α + effectors with increasing duration of co-infection (which was shorter in the earlier report): the relationship between *S. mansoni* and HIV disease is likely to be dynamic as HIV disease progresses. *S. mansoni* might hasten/enhance a general involutionary pattern (loss of polyfunctionality) seen in chronic HIV infection.

### T cell activation

T cell activation and proliferation contribute to productive HIV infection of memory CD4 T cells [25–28] and helminth infections have been associated with systemic T cell activation [29–31]. In addition, persistent immune activation is associated with progression to AIDS [32]. We found that HIV + SM + had a higher frequency of activated CD4 T cells than HIV + SM − participants. Our results concur with the study from Mkhize-Kwitshana et al., however, Mkhize-Kwitshana et al., suggest that the association of helminth infection with T cell activation may differ from one helminth species to another [33].

Bacterial lipopolysaccharide increases immune activation in simian human immunodeficiency virus-subtype B chronically infected macaques and alters T cell homeostasis [34]. A possible further contribution to immune activation in HIV-*S. mansoni* coinfection might be microbial product translocation, during the process of *S. mansoni* egg excretion resulting from disruption of gut epithelium [35, 36].

However, we found that the plasma viral load among the subset of participants who were tested for T cell activation was higher in the HIV + SM+ (mean pVL 68,980 RNA copies per ml for HIV + SM + and 43,704 RNA copies per ml for HIV + SM−; p > 5.0 × 10^− 03^), again raising questions as to causal direction: in HIV infection, the level of immune activation has been directly correlated to the plasma level of HIV [37].

### Th phenotype

It had been found that during HIV disease progression, the predominant anti HIV response changes from a predominant Th1 to a predominant Th2 profile [38, 39]. We hypothesized that *S. mansoni* infection might exacerbate this by directing the immune response towards another T helper profile: Th2, Th9, IL-10 producing CD4 T cells (Th10) or Th17. However, we found no evidence to this effect. In a mixed infection such as HIV-1 and *S. mansoni*, likely there is a balance among the different Th phenotypes with no predominant Th phenotype.

### CD8 T cell frequency and cytotoxic potential

The frequency of IFN-γ monofunctional CD8 T cells was higher in the HIV + SM + responders in this study, consistent with our earlier study in recently HIV infected individuals [24].

However, the most critical functional property of T cells from HIV infected elite controllers {HIV infected participants with no detectable plasma viral load despite being HIV infected for a very long time (> 10 years)}, has been reported to be their ability to inhibit viral replication in ex vivo infected autologous CD4 T cells [40, 41]. This inhibition has been attributed to the cytotoxic potential of T cells, which is mediated in part through cytotoxic granules such as perforin and granzymes. In elite controllers, the CD8 T cells synthesize greater amounts of cytotoxic granules such as perforin and granzymes [42–44]. These granules enhance the cytotoxic potential of such cells and can simultaneously induce apoptosis on target cells. Therefore, we tested whether *S. mansoni* was associated with cytotoxic potential of HIV specific CD8 T cells.

*S. mansoni* infection was associated with a lower frequency of HIV specific perforin producing CD8 T cells in HIV + SM+, compared with HIV + SM−. Similarly, McElroy et al. [45] also found a lower frequency of HIV specific CD8 T cells with CD107a (indicative of degranulation capability) in HIV + SM + compared to HIV + SM − participants. Thus, despite an increased frequency of IFN-γ + CD8 T cells, it is probable that *S. mansoni* co-infection is associated with impaired HIV-specific CD8 + T cell cytotoxic potential.

### HIV specific ADCC antibody responses

*S. mansoni* infection was associated with lower HIV specific ADCC-mediating antibody titre but not potency. However, there was no difference in envelope binding IgG1 and IgG3 antibody titre when compared to HIV + SM − participants (S9 and S10 Figs). This suggests that the ADCC mediators involved are likely not IgG1, IgG3 subclasses or IgG. It is also possible that the ADCC assay used in this study exposed different epitopes than those in the ELISA binding antibody assays. The A1953 infectious molecular clone that was used to coat the target cells was subtype B and most of the participants were infected with subtypes A1, D or C (based on gp41 sequencing and subtyping using REGA). Therefore, subtype differences could have accounted for the discordance between the Gran-ToxiLux assay and ELISA results. Epitope or antigen presentation in the ADCC assay could have induced conformational changes that exposed the ADCC inducing epitopes on the surface of the infected CEM.NKR CD4 T cell lines. The antigen in the ELISA assay was adsorbed to a plastic surface and therefore likely stationary.

### Summary

In summary, among HIV-positive adults, *S. mansoni* infection was associated with a range of differences in CD4 and CD8 T cell profiles, and with reduced ADCC antibody titres, suggesting that HIV specific immunity is altered, probably impaired, during *S. mansoni* coinfection. Our results stress the importance of understanding the impact of *S. mansoni* treatment upon HIV-specific immune responses and disease progression. The participants in this study have been followed up to address this matter.

The study was conducted between August 2012 and September 2015. During this time, the government policy was that only individuals with CD4 count below 350 CD4 cells per µl of blood were eligible for ART. However, this policy has since changed and any individual who tests HIV positive is eligible for ART irrespective of CD4 count. Although the policy was put in place, not all individuals living with HIV are on ART. Fishing communities have challenges of access to health services including provision of ART and HIV care services such as frequent drug stock outs for extended periods, mobility and transport challenges [7, 46]. Based on the inadequate health care services in the fishing communities, the outcome of the results from the study is significant in describing the pathogenesis of people living with HIV in Uganda.

### Electronic supplementary material

Below is the link to the electronic supplementary material.

**Additional File 1:** Gating strategy used to analyse SIS PBMC samples stained with Th1 flow cytometry panel. PBMC were stimulated with 1?g/ml of GAG PTE POOL-1 for 16-18 hours in presence of Golgiplug. The single cytokine responses shown on the diagram were then Boolean gated.

**Additional File 2:** Gating strategy used to analyse SIS PBMC samples stained with Th1/Th2/Th9/IL-10 producing CD4 T cells/Th17 panel flow cytometry panel. PBMC were stimulated with 200ng/ml of SEB for 17 hours in presence of Golgiplug.

**Additional File 3:** Gating strategy used to analyse SIS PBMC samples stained with positive and negative regulatory receptors T cell flow cytometry panel. PBMC were not stimulated.

**Additional File 4:** Gating strategy used to analyse SIS PBMC samples stained with cytotoxic potential flow cytometry panel. PBMC were stimulated with 1?g/ml of GAG PTE POOL-1 for 17 hours in presence of 1 ?l of Golgiplug and 1 ?l of Golgistop. IFN-? was introduced into the panel to as a surrogate for HIV specific CD8 T cells.

**Additional File 5:** Gating strategy used to analyse SIS PBMC samples stained with regulatory T cell flow cytometry panel. PBMC were not stimulated.

**Additional File 6:** Polyfunctionality of CD8 T cell cytokine responses after GAG PTE POOL-1 stimulation. Same legend as for Fig. 1, showing HIV+SM+ (n=14) and HIV+SM? (n=14) responders.

**Additional File 7:** Ratio of frequency of IL-10 producing CD4 T cells, Th2, Th9 and Th17 to Th1 in response to (A) p24, (B) GAG PTE POOL-1, (C) GAG PTE POOL 2 and (D) SEB stimulations. p24 HIV+SM+ n= 5 HIV+SM? n=4, GAG PTE POOL-1 HIV+SM+ n= 9 HIV+SM? n=5, GAG PTE POOL-2 HIV+SM+ n= 9 HIV+SM? n=5 and SEB HIV+SM+ n= 14 HIV+SM? n=10. Comparison of ratio of IL-10 producing CD4 T cells, Th2, Th9 and Th17 to Th1 between HIV+SM+ and HIV+SM?. Student?s t test and the Holm-S?d?k correction for multiple comparisons were used to compare the response to each stimulant between HIV+SM+ and HIV+SM?. No significant p values were observed. The horizontal line shows the mean while the vertical lines shows the 95% confidence interval. The error bars not shown are clipped at the axis.

**Additional File 8:** Association of activated T reg and S. mansoni infection in HIV+SM+. Comparison of activated CD4 regulatory T cells between HIV+SM+ (n=18) and HIV+SM? (n=15). The mean frequency of activated CD4 regulatory T cells was compared using Student?s t test. The p value was >0.05. The horizontal line shows the mean and the vertical line shows the 95% confidence interval.

**Additional File 9:** Association of IgG1 titres and S. mansoni infection in HIV+SM+. The IgG1 was binding to gp41 MN (A), gp140 UG21 (B), gp140 UG37 (C) and gp140 SF (D) antigen. One-way ANOVA with Dunnett multiple comparison test correction was used to compare mean IgG1 titres between HIV+SM+ (n=15) and HIV+SM? (n=15). The horizontal line shows the mean and the vertical line shows the 95% confidence interval.

**Additional File 10:** Association of IgG3 titres and S. mansoni infection in HIV+SM+. The IgG1 was binding to gp41 MN (A), gp140 UG21 (B), gp140 UG37 (C) and gp140 SF (D) antigen. One-way ANOVA with Dunnett multiple comparison test correction was used to compare mean IgG1 titres between HIV+SM+ (n=15) and HIV+SM? (n=15). The horizontal line shows the mean and the vertical line shows the 95% confidence interval.

**Additional File 11:** Response rates

**Additional File 12:** CD8 Mean median Fluorescence Intensity (MFI)

**Additional File 13:** Shows the frequency of CD4 (a) and CD8 (b) T cells expressing inhibitory and stimulatory receptors

## Data Availability

The datasets generated and/or analysed during the current study are not publicly available but are available from the corresponding author on reasonable request.

## List of Abbreviation

ADCC: Antibody-dependent cell-mediated cytotoxic
AIDS: Acquired Immunodeficiency syndrome
ART: Antiretroviral therapy
GAG: Group specific antigen
HIV: Human Immunodeficiency Virus
HIV + SM +: HIV and *S.mansoni* coinfected individual
HIV + SM −: HIV infected and *S.mansoni* uninfected individual
HRP: Horseradish peroxidase enzyme
IFNγ: Interferon gamma
IL-2: Interleukin 2
IL-4: Interleukin4
IL-5: Interleukin 5
IL-9: Interleukin 9
IL-10: Interleukin 10
IL-13: Interleukin 13
KK: Kato Katz
MFI: Median Fluorescence Intensity
NFL1: Viability marker
PBMC: Peripheral blood mononuclear cells
PTE: potential T cell epitopes
PBST: (PBS with 0.03% Tween 20)
PBS: Phosphate buffered saline
PZQ: praziquantel
RNA: Ribonucleic acid
SIS: Schistosomiasis Intervention Study
SEB: Staphylococcus enterotoxin B
*S. mansoni*: Schistosoma Mansoni
Th1: T helper 1
Th2: T helper 2
Th9: T helper 9
Th10: T helper 10
Th17: T helper 17
Th22: T helper 22
T regs: Regulatory T cells
TNF-α: Tumor Necrosis Factor
TFL4: fluorescent target cell marker
%GzB: %Granzyme B.
