# Supplementary figures and images for: HIV specific Th1 responses are altered in Ugandans with HIV and Schistosoma mansoni coinfection
Source: BMC Immunol. 2023 Aug 29;24:25. doi: 10.1186/s12865-023-00554-3 (PMC10466713; doi:10.1186/s12865-023-00554-3)

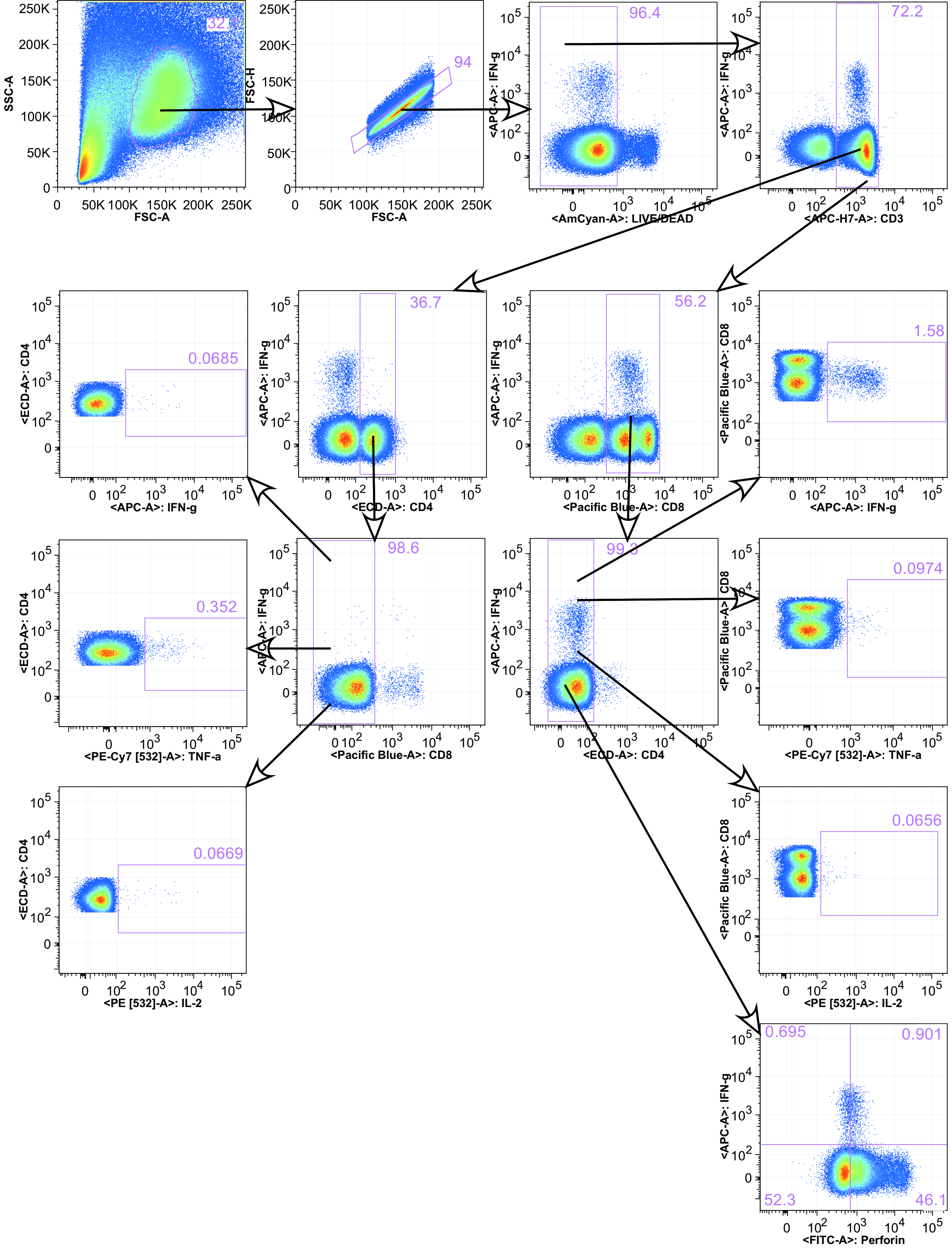

Supplement: Supplementary file 1 — Additional File 1: Gating strategy used to analyse SIS PBMC samples stained with Th1 flow cytometry panel. PBMC were stimulated with 1?g/ml of GAG PTE POOL-1 for 16-18 hours in presence of Golgiplug. The single cytokine responses shown on the diagram were then Boolean gated. [file 12865_2023_554_MOESM1_ESM.tiff]

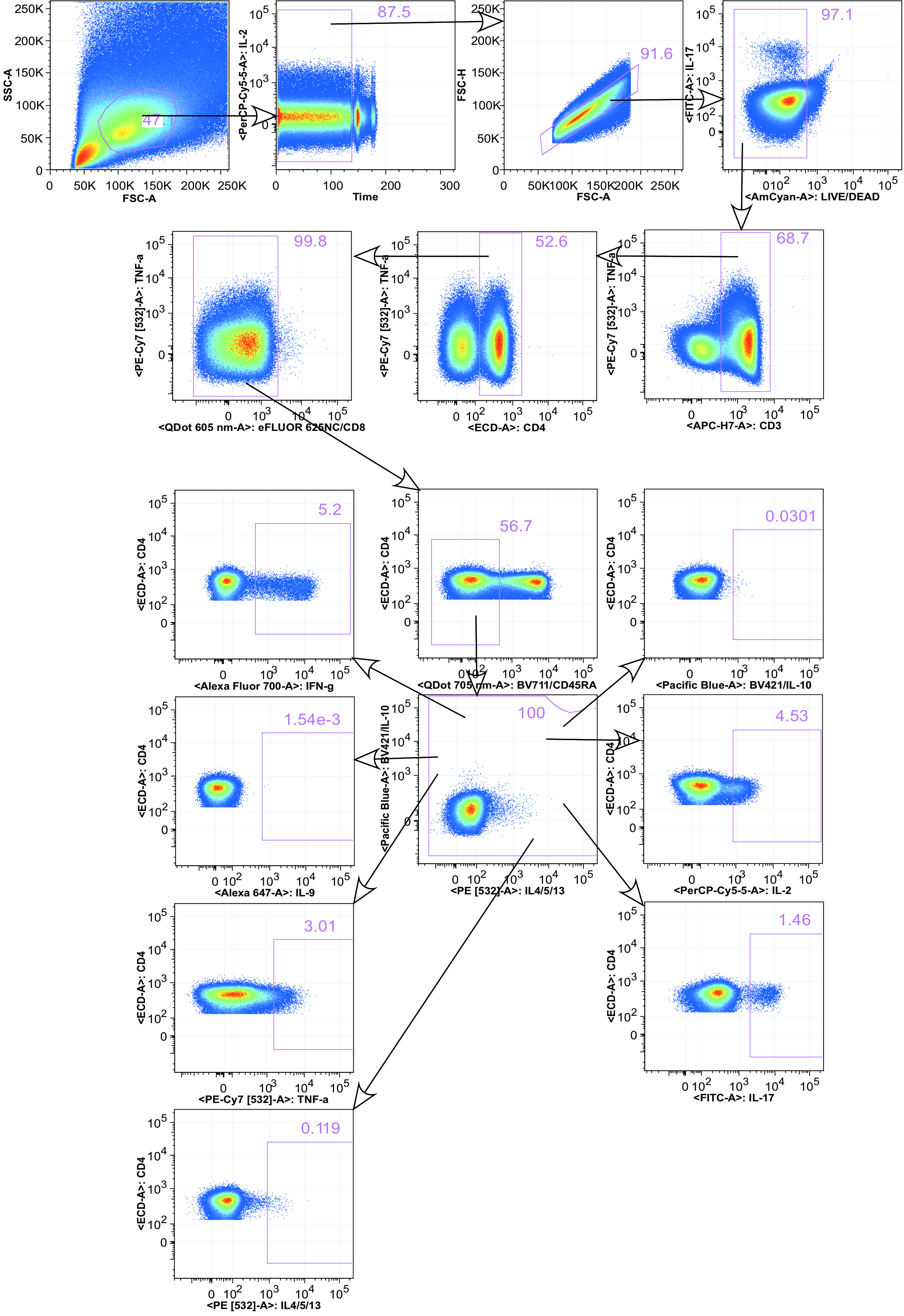

Supplement: Supplementary file 2 — Additional File 2: Gating strategy used to analyse SIS PBMC samples stained with Th1/Th2/Th9/IL-10 producing CD4 T cells/Th17 panel flow cytometry panel. PBMC were stimulated with 200ng/ml of SEB for 17 hours in presence of Golgiplug. [file 12865_2023_554_MOESM2_ESM.tiff]

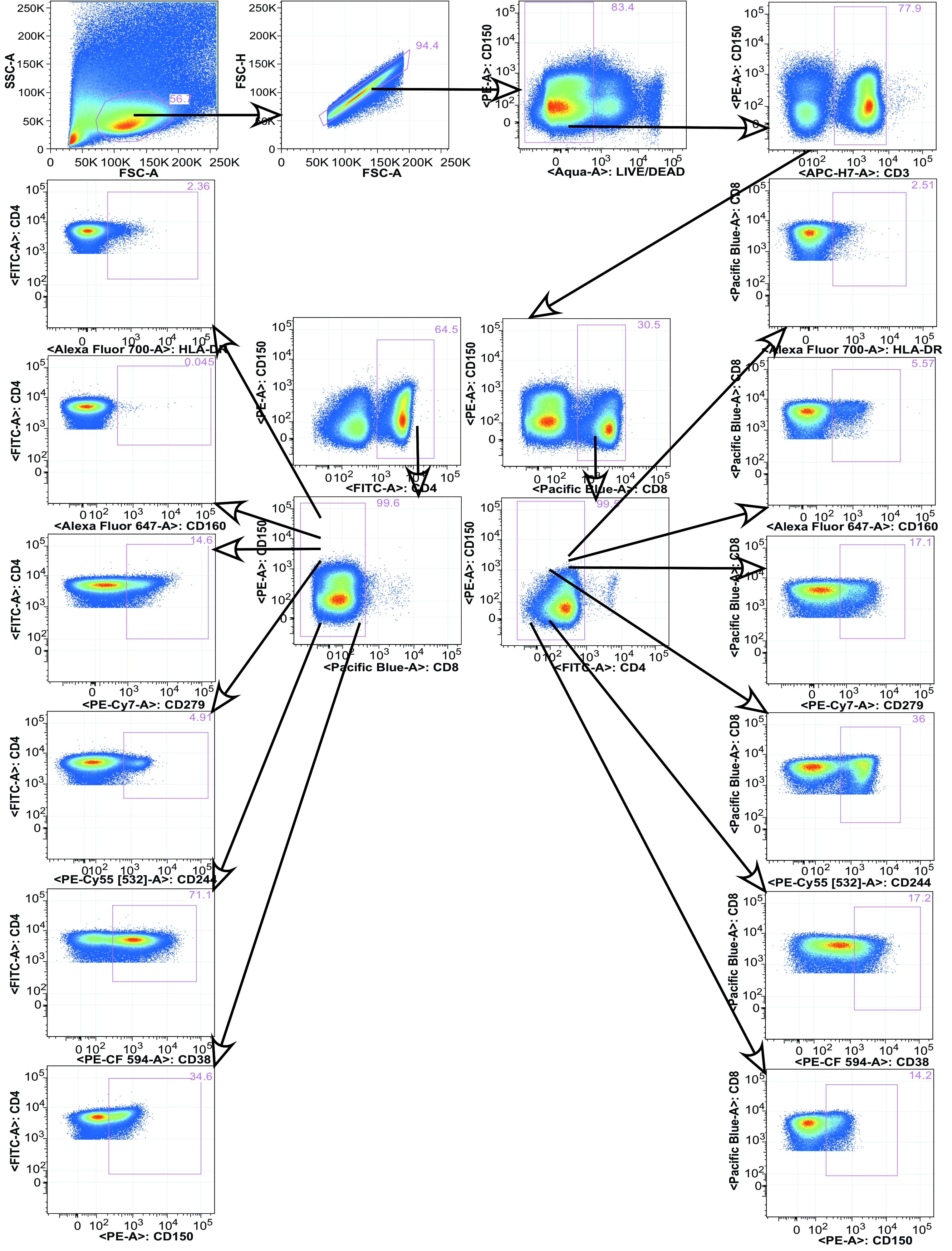

Supplement: Supplementary file 3 — Additional File 3: Gating strategy used to analyse SIS PBMC samples stained with positive and negative regulatory receptors T cell flow cytometry panel. PBMC were not stimulated. [file 12865_2023_554_MOESM3_ESM.tiff]

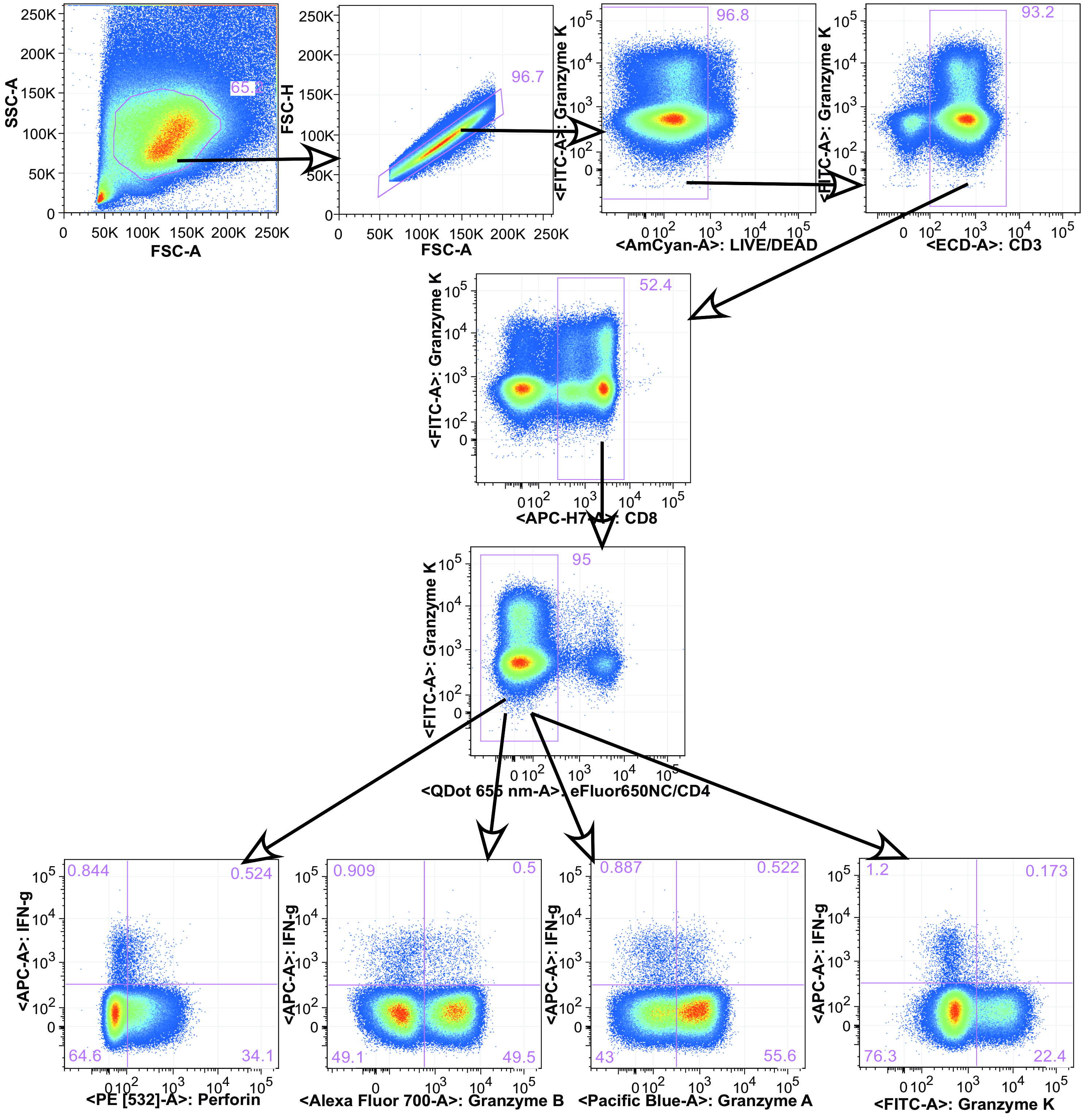

Supplement: Supplementary file 4 — Additional File 4: Gating strategy used to analyse SIS PBMC samples stained with cytotoxic potential flow cytometry panel. PBMC were stimulated with 1?g/ml of GAG PTE POOL-1 for 17 hours in presence of 1 ?l of Golgiplug and 1 ?l of Golgistop. IFN-? was introduced into the panel to as a surrogate for HIV specific CD8 T cells. [file 12865_2023_554_MOESM4_ESM.tiff]

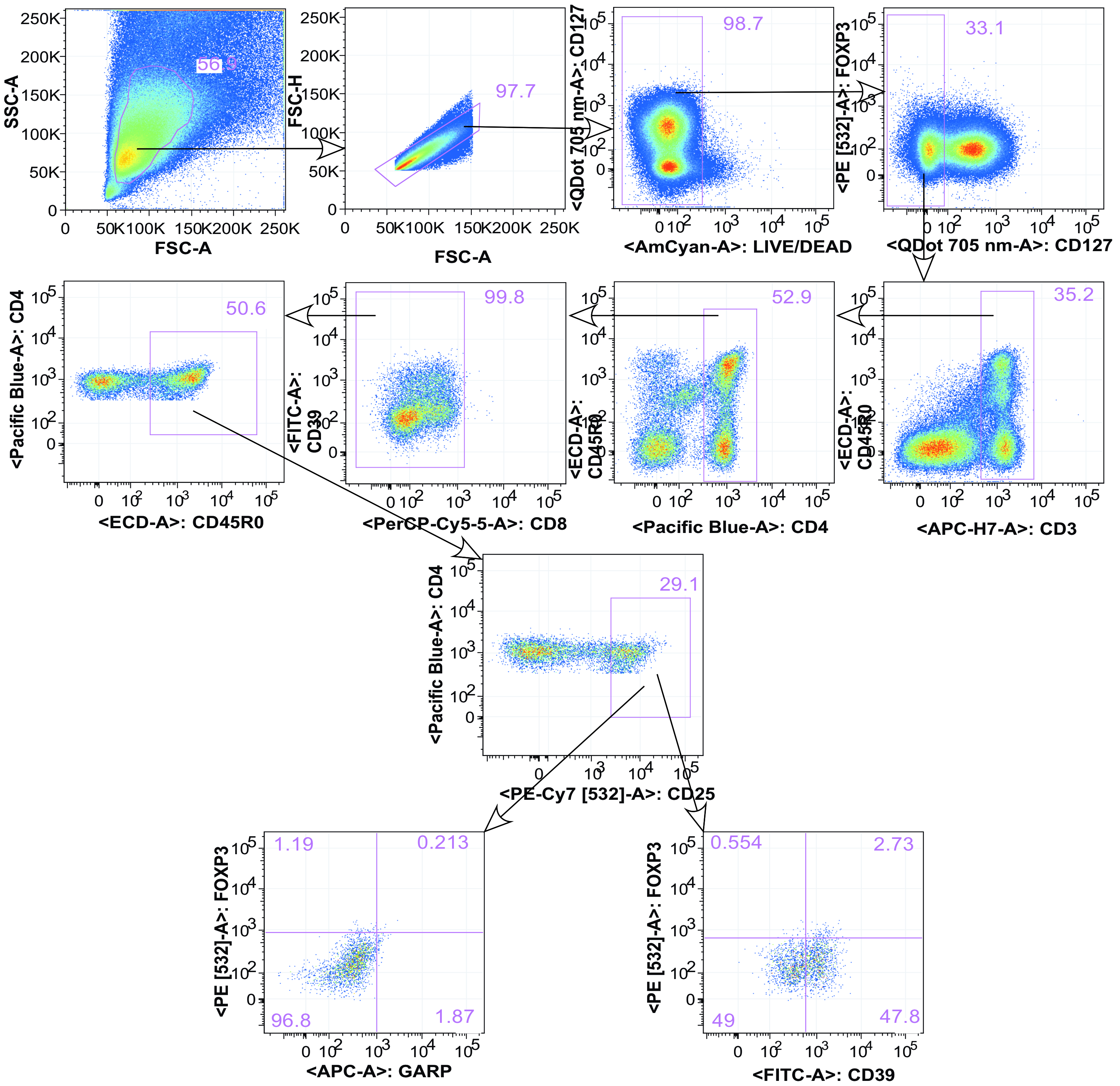

Supplement: Supplementary file 5 — Additional File 5: Gating strategy used to analyse SIS PBMC samples stained with regulatory T cell flow cytometry panel. PBMC were not stimulated. [file 12865_2023_554_MOESM5_ESM.tiff]

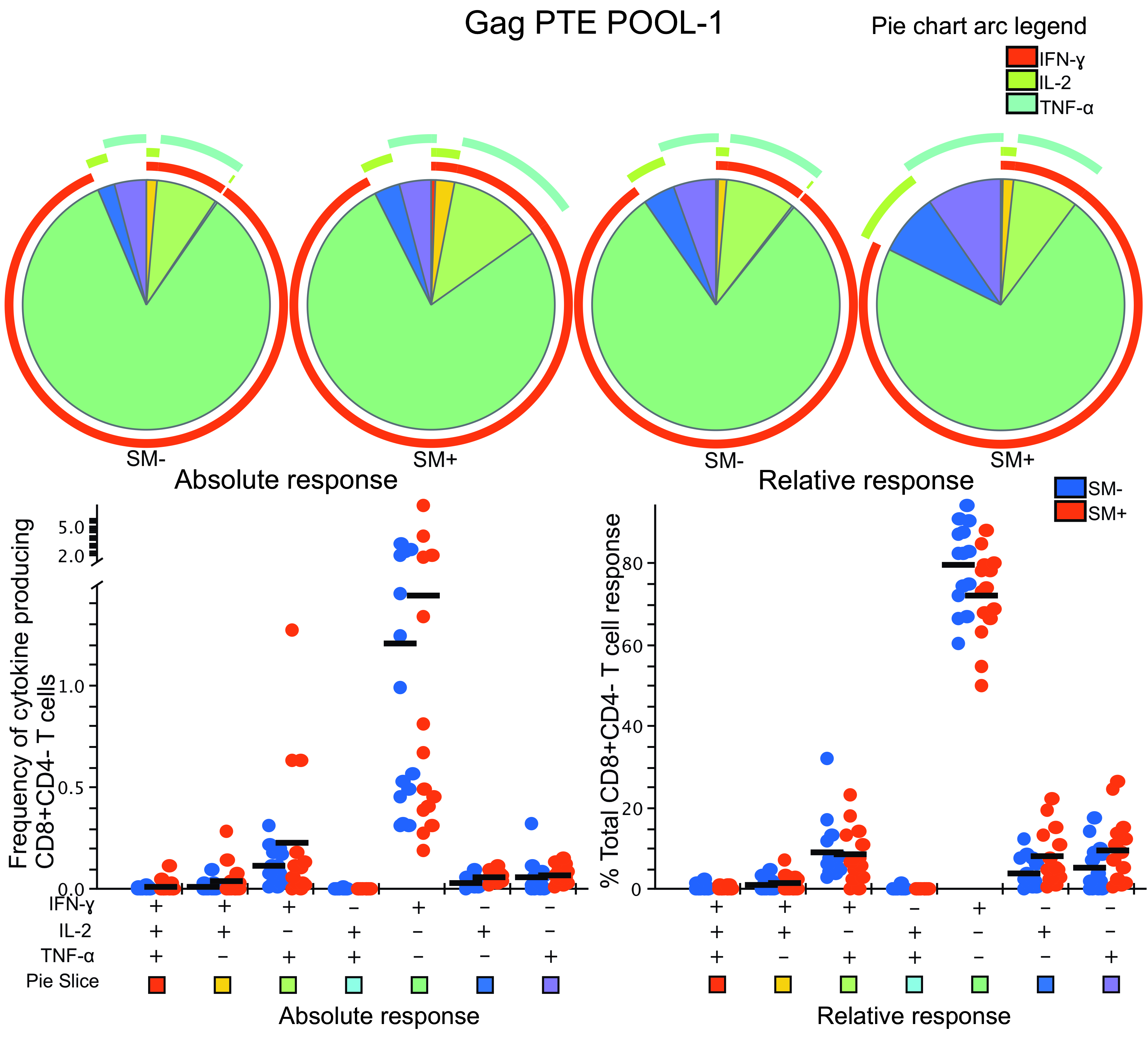

Supplement: Supplementary file 6 — Additional File 6: Polyfunctionality of CD8 T cell cytokine responses after GAG PTE POOL-1 stimulation. Same legend as for Fig. 1, showing HIV+SM+ (n=14) and HIV+SM? (n=14) responders. [file 12865_2023_554_MOESM6_ESM.tiff]

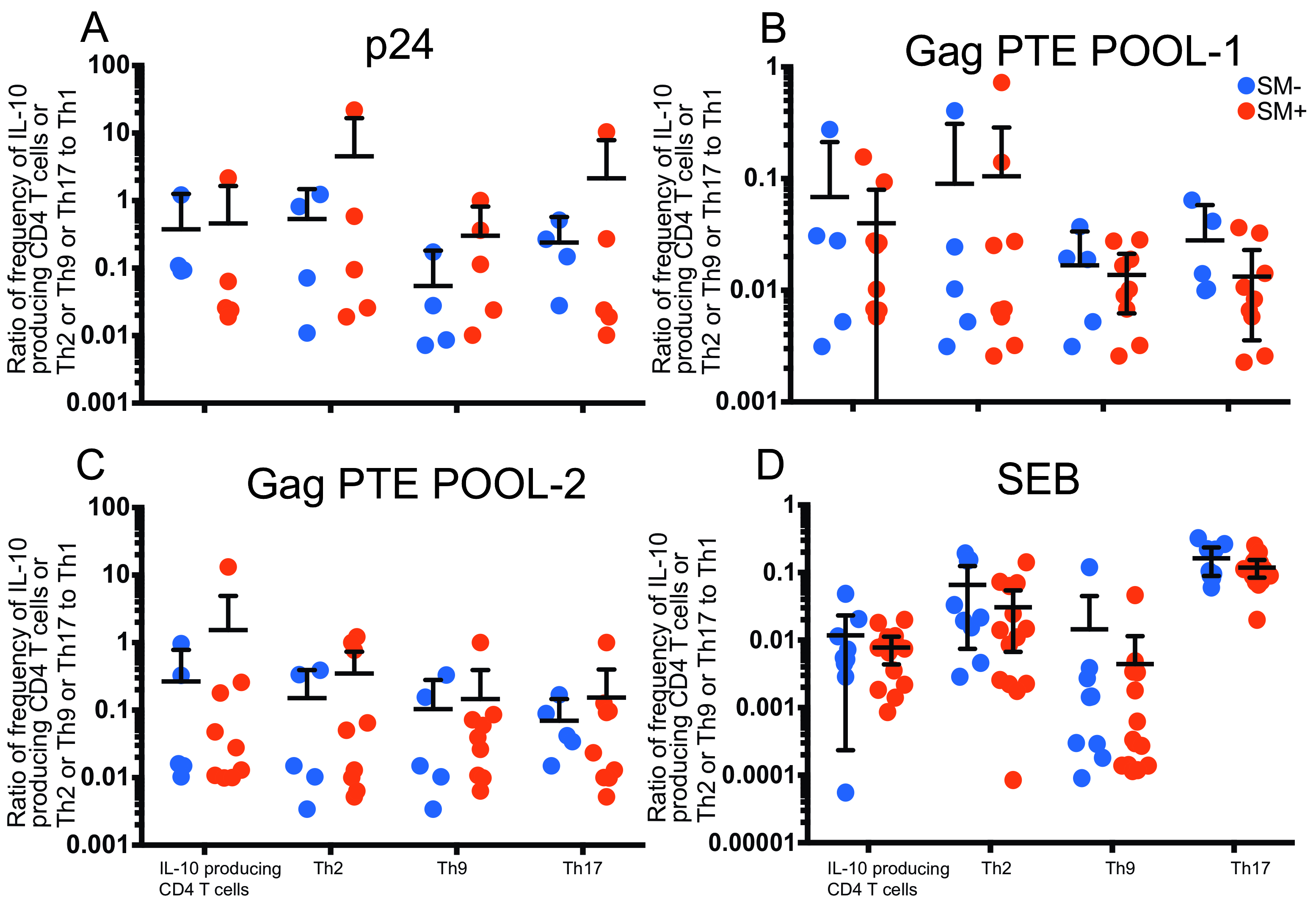

Supplement: Supplementary file 7 — Additional File 7: Ratio of frequency of IL-10 producing CD4 T cells, Th2, Th9 and Th17 to Th1 in response to (A) p24, (B) GAG PTE POOL-1, (C) GAG PTE POOL 2 and (D) SEB stimulations. p24 HIV+SM+ n= 5 HIV+SM? n=4, GAG PTE POOL-1 HIV+SM+ n= 9 HIV+SM? n=5, GAG PTE POOL-2 HIV+SM+ n= 9 HIV+SM? n=5 and SEB HIV+SM+ n= 14 HIV+SM? n=10. Comparison of ratio of IL-10 producing CD4 T cells, Th2, Th9 and Th17 to Th1 between HIV+SM+ and HIV+SM?. Student?s t test and the Holm-S?d?k correction for multiple comparisons were used to compare the response to each stimulant between HIV+SM+ and HIV+SM?. No significant p values were observed. The horizontal line shows the mean while the vertical lines shows the 95% confidence interval. The error bars not shown are clipped at the axis. [file 12865_2023_554_MOESM7_ESM.tiff]

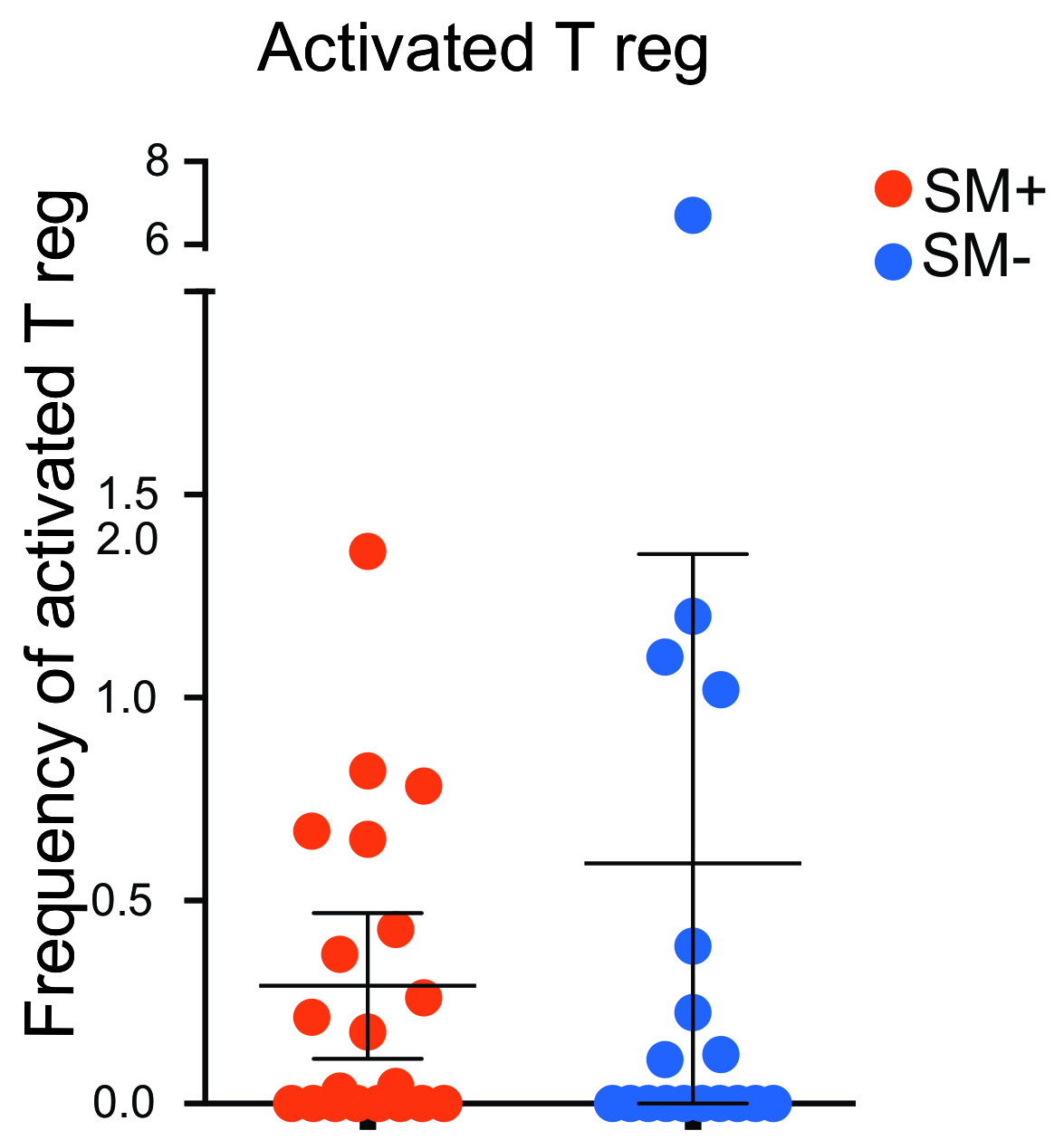

Supplement: Supplementary file 8 — Additional File 8: Association of activated T reg and S. mansoni infection in HIV+SM+. Comparison of activated CD4 regulatory T cells between HIV+SM+ (n=18) and HIV+SM? (n=15). The mean frequency of activated CD4 regulatory T cells was compared using Student?s t test. The p value was >0.05. The horizontal line shows the mean and the vertical line shows the 95% confidence interval. [file 12865_2023_554_MOESM8_ESM.tiff]

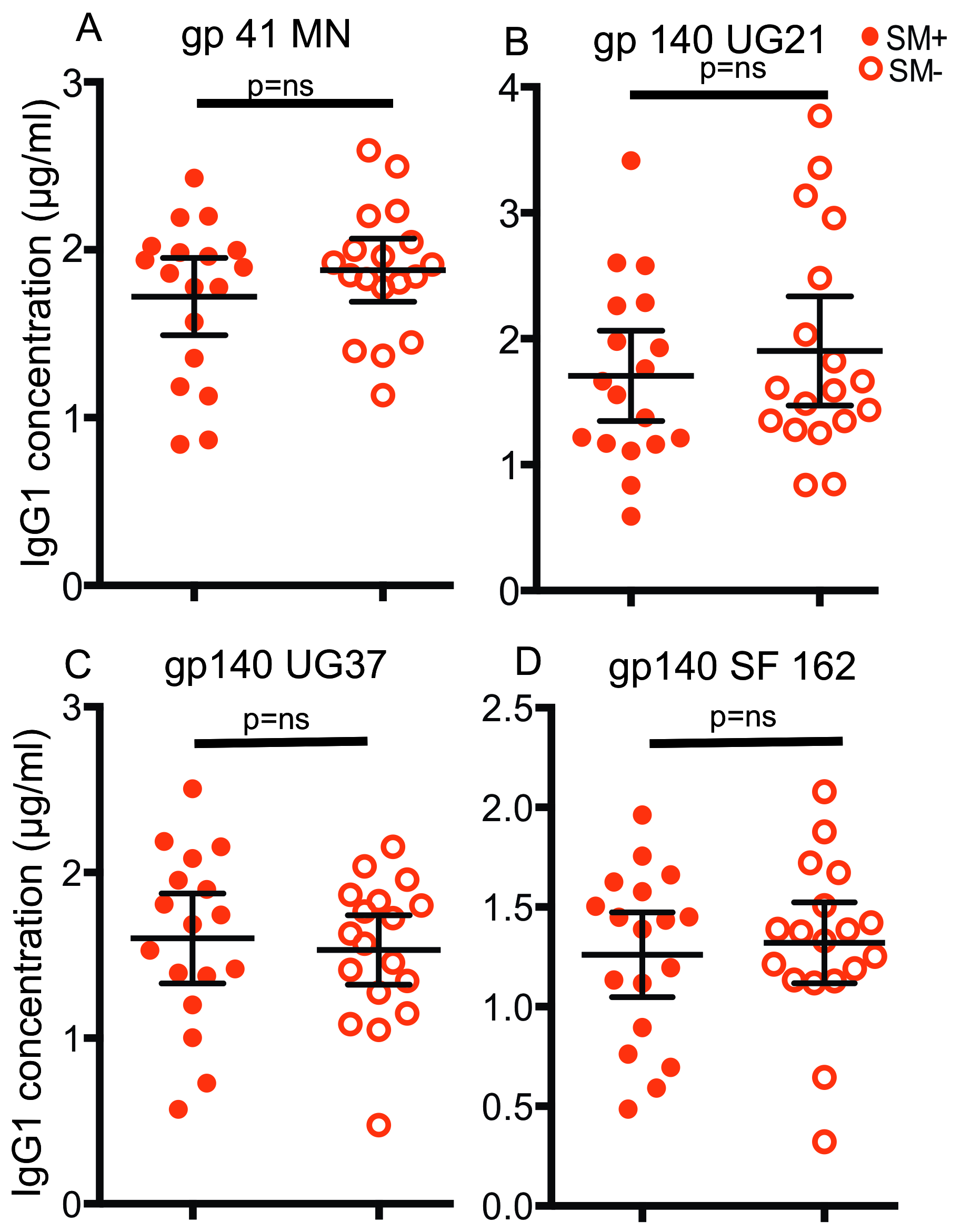

Supplement: Supplementary file 9 — Additional File 9: Association of IgG1 titres and S. mansoni infection in HIV+SM+. The IgG1 was binding to gp41 MN (A), gp140 UG21 (B), gp140 UG37 (C) and gp140 SF (D) antigen. One-way ANOVA with Dunnett multiple comparison test correction was used to compare mean IgG1 titres between HIV+SM+ (n=15) and HIV+SM? (n=15). The horizontal line shows the mean and the vertical line shows the 95% confidence interval. [file 12865_2023_554_MOESM9_ESM.tiff]

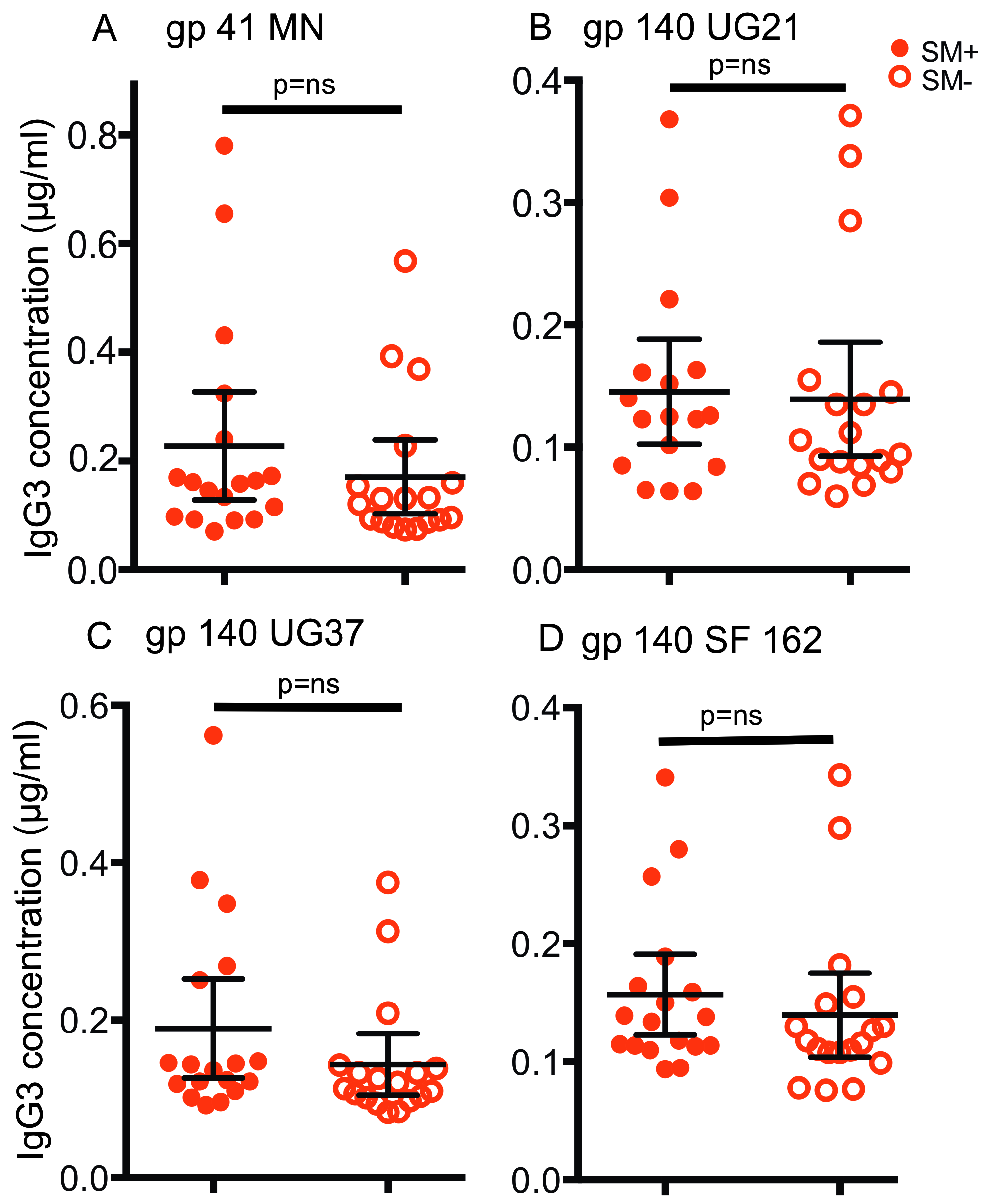

Supplement: Supplementary file 10 — Additional File 10: Association of IgG3 titres and S. mansoni infection in HIV+SM+. The IgG1 was binding to gp41 MN (A), gp140 UG21 (B), gp140 UG37 (C) and gp140 SF (D) antigen. One-way ANOVA with Dunnett multiple comparison test correction was used to compare mean IgG1 titres between HIV+SM+ (n=15) and HIV+SM? (n=15). The horizontal line shows the mean and the vertical line shows the 95% confidence interval. [file 12865_2023_554_MOESM10_ESM.tiff]
