# Supplementary material for: HIV specific Th1 responses are altered in Ugandans with HIV and Schistosoma mansoni coinfection
Source: BMC Immunol. 2023 Aug 29;24:25. doi: 10.1186/s12865-023-00554-3 (PMC10466713; doi:10.1186/s12865-023-00554-3)
Supplement: Supplementary file 11 — Additional File 11: Response rates [file 12865_2023_554_MOESM11_ESM.docx]

S1 Table. Response rates

| Stimulus | Number of SM− participants stimulated | Number of SM− responders | Number of SM+ participants stimulated | Number of SM+ responders | p-value* | T cell subset |
| --- | --- | --- | --- | --- | --- | --- |
| GAG PTE POOL-1 | 16 | 15 | 16 | 16 | 1.00 | CD4 |
| GAG PTE POOL-2 | 16 | 13 | 16 | 14 | 1.00 | CD4 |
| GAG PTE POOL-1 | 16 | 14 | 16 | 14 | 1.00 | CD8 |
| GAG PTE POOL-2 | 16 | 11 | 16 | 11 | 1.00 | CD8 |

* The number of responders to non responders was compared using Fishers exact test.
