# Supplementary material for: HIV specific Th1 responses are altered in Ugandans with HIV and Schistosoma mansoni coinfection
Source: BMC Immunol. 2023 Aug 29;24:25. doi: 10.1186/s12865-023-00554-3 (PMC10466713; doi:10.1186/s12865-023-00554-3)
Supplement: Supplementary file 12 — Additional File 12: CD8 Mean median Fluorescence Intensity (MFI) [file 12865_2023_554_MOESM12_ESM.docx]

# Supplementary figure captions

S1 Fig. Gating strategy used to analyse SIS PBMC samples stained with Th1 flow cytometry panel.

PBMC were stimulated with 1μg/ml of GAG PTE POOL-1 for 16-18 hours in presence of Golgiplug. The single cytokine responses shown on the diagram were then Boolean gated.

S2 Fig. Gating strategy used to analyse SIS PBMC samples stained with Th1/Th2/Th9/IL-10 producing CD4 T cells/Th17 panel flow cytometry panel.

PBMC were stimulated with 200ng/ml of SEB for 17 hours in presence of Golgiplug.

S3 Fig. Gating strategy used to analyse SIS PBMC samples stained with positive and negative regulatory receptors T cell flow cytometry panel.

PBMC were not stimulated.

S4 Fig. Gating strategy used to analyse SIS PBMC samples stained with cytotoxic potential flow cytometry panel.

PBMC were stimulated with 1μg/ml of GAG PTE POOL-1 for 17 hours in presence of 1 μl of Golgiplug and 1 μl of Golgistop. IFN-γ was introduced into the panel to as a surrogate for HIV specific CD8 T cells.

S5 Fig. Gating strategy used to analyse SIS PBMC samples stained with regulatory T cell flow cytometry panel.

PBMC were not stimulated.

S6 Fig. Polyfunctionality of CD8 T cell cytokine responses after GAG PTE POOL-1 stimulation. Same legend as for Fig. 1, showing HIV+SM+ (n=14) and HIV+SM− (n=14) responders.

S7 Fig. Ratio of frequency of IL-10 producing CD4 T cells, Th2, Th9 and Th17 to Th1 in response to (A) p24, (B) GAG PTE POOL-1, (C) GAG PTE POOL 2 and (D) SEB stimulations.

p24 HIV+SM+ n= 5 HIV+SM− n=4, GAG PTE POOL-1 HIV+SM+ n= 9 HIV+SM− n=5, GAG PTE POOL-2 HIV+SM+ n= 9 HIV+SM− n=5 and SEB HIV+SM+ n= 14 HIV+SM− n=10.

Comparison of ratio of IL-10 producing CD4 T cells, Th2, Th9 and Th17 to Th1 between HIV+SM+ and HIV+SM−. Student’s t test and the Holm-Sídák correction for multiple comparisons were used to compare the response to each stimulant between HIV+SM+ and HIV+SM−. No significant p values were observed. The horizontal line shows the mean while the vertical lines shows the 95% confidence interval. The error bars not shown are clipped at the axis.

S8 Fig. Association of activated T reg and *S. mansoni* infection in HIV+SM+. Comparison of activated CD4 regulatory T cells between HIV+SM+ (n=18) and HIV+SM− (n=15). The mean frequency of activated CD4 regulatory T cells was compared using Student’s t test. The p value was >0.05. The horizontal line shows the mean and the vertical line shows the 95% confidence interval.

S9 Fig. Association of IgG1 titres and *S. mansoni* infection in HIV+SM+. The IgG1 was binding to gp41 MN (A), gp140 UG21 (B), gp140 UG37 (C) and gp140 SF (D) antigen. One-way ANOVA with Dunnett multiple comparison test correction was used to compare mean IgG1 titres between HIV+SM+ (n=15) and HIV+SM− (n=15). The horizontal line shows the mean and the vertical line shows the 95% confidence interval.

S10 Fig. Association of IgG3 titres and *S. mansoni* infection in HIV+SM+. The IgG1 was binding to gp41 MN (A), gp140 UG21 (B), gp140 UG37 (C) and gp140 SF (D) antigen. One-way ANOVA with Dunnett multiple comparison test correction was used to compare mean IgG1 titres between HIV+SM+ (n=15) and HIV+SM− (n=15). The horizontal line shows the mean and the vertical line shows the 95% confidence interval.
