# Supplementary material for: HIV specific Th1 responses are altered in Ugandans with HIV and Schistosoma mansoni coinfection
Source: BMC Immunol. 2023 Aug 29;24:25. doi: 10.1186/s12865-023-00554-3 (PMC10466713; doi:10.1186/s12865-023-00554-3)
Supplement: Supplementary file 13 — Additional File 13: Shows the frequency of CD4 (a) and CD8 (b) T cells expressing inhibitory and stimulatory receptors [file 12865_2023_554_MOESM13_ESM.docx]

S2 Table . CD8 Mean median Fluorescence Intensity (MFI)

| Cytokine | Mean MFI SM− (n) | Mean MFI SM+ (n) | p-value | Stimulant |
| --- | --- | --- | --- | --- |
| IFN-γ | 1146 (14) | 1847 (14) | 7.1x10^-02^ | GAG PTE POOL-1 |
| IL-2 | 167 (14) | 167 (14) | 9.8x10^-01^ | GAG PTE POOL-1 |
| TNF-α | 1210 (14) | 1059 (14) | 2.9x10^-01^ | GAG PTE POOL-1 |
| IFN-γ | 886 (11) | 1317 (11) | 3.2x10^-01^ | GAG PTE POOL-2 |
| IL-2 | 185 (11) | 178 (11) | 6.9x10^-01^ | GAG PTE POOL-2 |
| TNF-α | 1145 (11) | 1025 (11) | 4.7x10^-01^ | GAG PTE POOL-2 |
